# Supplementary material for: Sarm1 haploinsufficiency or low expression levels after antisense oligonucleotides delay programmed axon degeneration
Source: Cell Rep. 2021 Dec 14;37(11):110108. doi: 10.1016/j.celrep.2021.110108 (PMC8692746; doi:10.1016/j.celrep.2021.110108)
Supplement: Document S1. Figures S1–S3 [file mmc1.pdf]

Cell Reports, Volume *37*

Supplemental information

Sarm1 haploinsufficiency or low expression levels after antisense oligonucleotides delay programmed axon degeneration

Stacey Anne Gould, Jonathan Gilley, Karen Ling, Paymaan Jafar-Nejad, Frank Rigo, and Michael Coleman

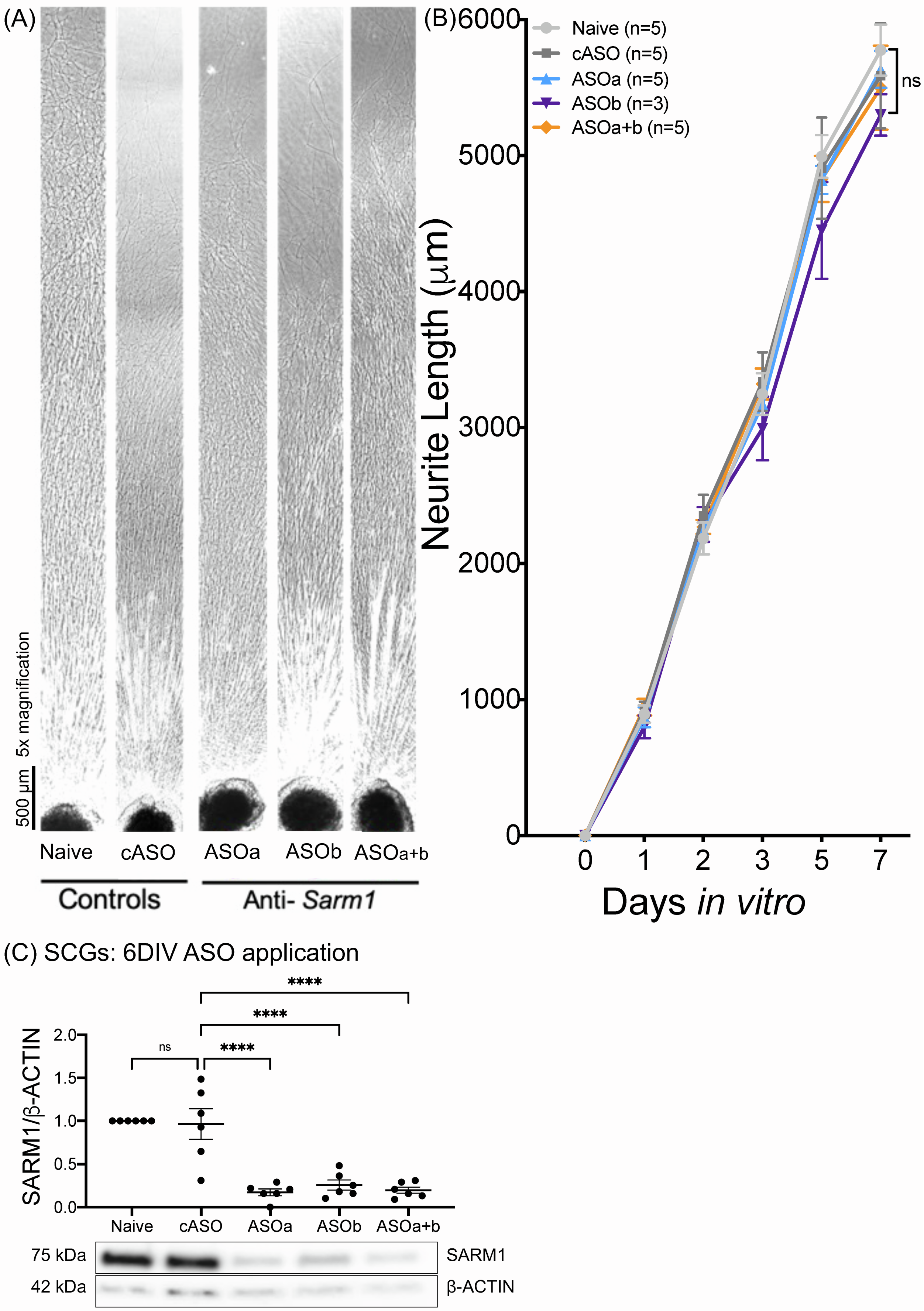
(A)
5x magnification
500 µm
Naive
cASO
ASOa
ASOb
ASOa+b
Controls
Anti- Sarm1
(B)
Naive (n=5)
cASO (n=5)
ASOa (n=5)
ASOb (n=3)
ASOa+b (n=5)
ns
Neurite Length (µm)
6000
5000
4000
3000
2000
1000
0
0
1
2
3
5
7
Days in vitro
(C) SCGs: 6DIV ASO application
****
****
ns
****
SARM1/β-ACTIN
2.0
1.5
1.0
0.5
0.0
Naive
cASO
ASOa
ASOb
ASOa+b
75 kDa
42 kDa
SARM1
β-ACTIN

Supplemental Figure 1: *Sarm1* antisense oligonucleotides do not affect SCG neurite outgrowth and significantly decrease SARM1 protein levels. Extra controls related to Figures 4 and 5. Representative phase contrast images of full length (5x magnification) SCG explants from P0 mice (a). Quantification of wild-type neurite outgrowth in the presence of plain media (naïve), cASO, ASOa, ASOb, or combined ASOa+b across seven days *in vitro* (b). ASOs were applied the day after plating (day1). Western blot shows that SARM1 band intensity is lower in *Sarm1* ASO-treated SCG cultured for 7DIV than cASO-treated SCGs when normalised to β-ACTIN; representative bands are shown below the quantification (c). A two-way ANOVA was performed followed by Dunnett's post-hoc analysis to determine if there was a significant difference in neurite outgrowth between cASO- and *Sarm1* ASO-treated SCGs. A one-way ANOVA was performed followed by Dunnett's post-hoc analysis to determine whether there was a significant difference in SARM1 band intensity between cASO-treated SCGs and *Sarm1* ASO-treated SCGs. ****$p<0.0001$; ns=not significant. Sample sizes for outgrowth and Western blot were from 3-5 mice per condition. Each data point represents an individual mouse where both SCGs were cultured in the presence of ASOs, or the average neurite length from three fields of view from two SCGs cultured together from one mouse. Data are presented as mean ± SEM. Scale bar represents 500 µm.

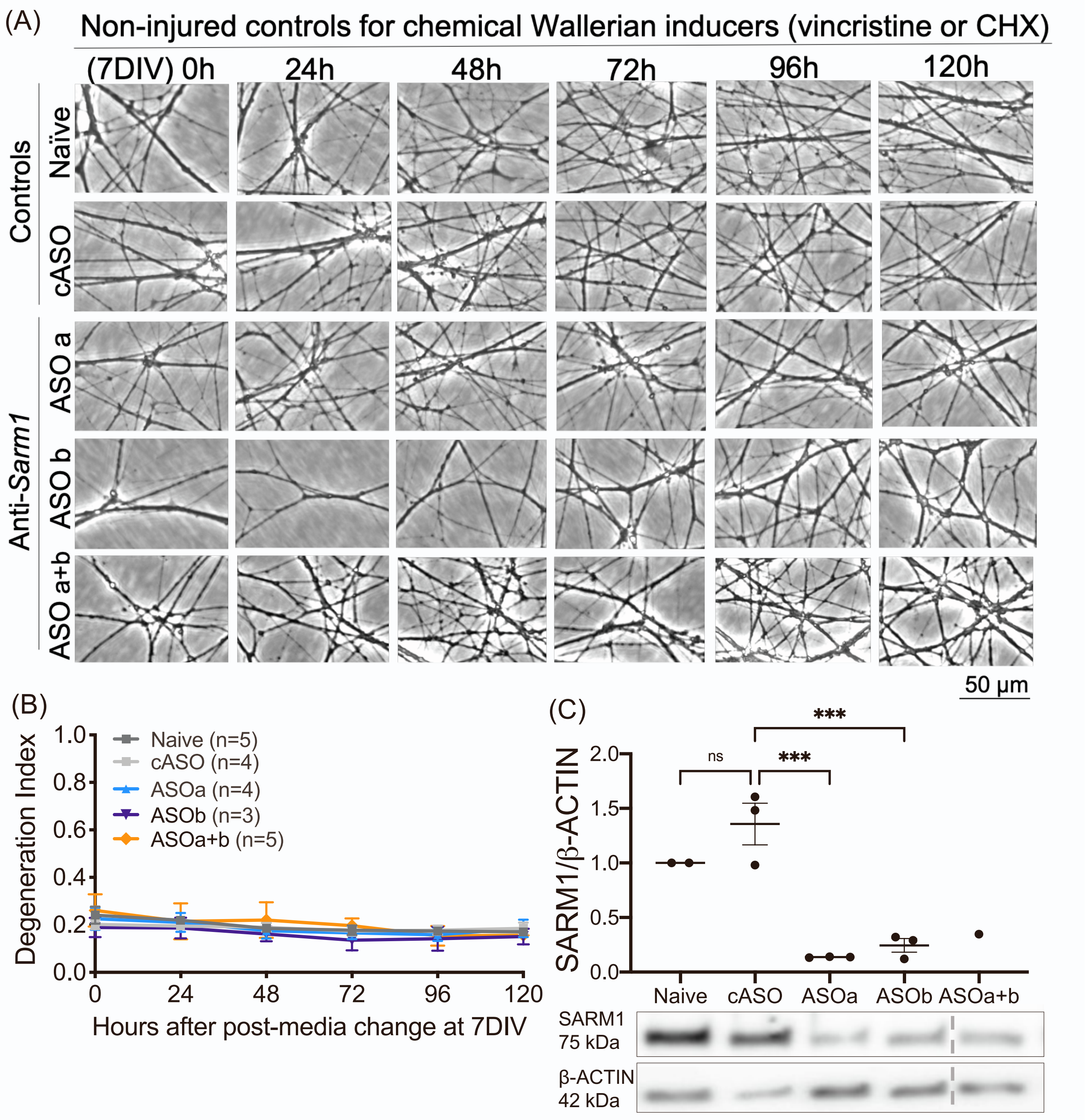
(A)
Non-injured controls for chemical Wallerian inducers (vincristine or CHX)
(7DIV) 0h
24h
48h
72h
96h
120h
Controls
Naïve
cASO
Anti-Sarm1
ASO a
ASO b
ASO a+b
50 μm
(B)
Degeneration Index
Naive (n=5)
cASO (n=4)
ASOa (n=4)
ASOb (n=3)
ASOa+b (n=5)
Hours after post-media change at 7DIV
(C)
SARM1/β-ACTIN
ns
***
***
Naive
cASO
ASOa
ASOb
ASOa+b
SARM1
75 kDa
β-ACTIN
42 kDa

Supplemental Figure 2: Prolonged exposure to *Sarm1* antisense oligonucleotides does not impair axon morphology or further lower SARM1 levels. Extra controls related to Figure 5. Representative phase contrast images of distal SCG neurites (20x magnification) from P0 mice (a) and quantification of degeneration index (b). SCGs were grown for 12DIV with ASOs applied for 11DIV (starting the day after plating). At 7DIV (0h), chemical inducers of programmed axon degeneration were applied to experimental groups, but plain media containing ASOs were applied to this cohort to confirm that prolonged exposure did not cause spontaneous degeneration or further lower SARM1 levels. Western blot analysis shows that SARM1 levels are lower in *Sarm1* ASO-treated SCGs than cASO-treated SCGs when normalised to β-ACTIN, but no further decreased than after 6DIV application (compare with S2); representative bands are shown below the quantification; grey line indicates sample was in a non-adjacent well on the same blot (c). A one-way ANOVA was performed followed by Dunnett's post-hoc analysis to determine whether there was a significant difference in SARM1 band intensity between cASO-treated SCGs and *Sarm1* ASO-treated SCGs. ***$p<0.001$. Sample sizes for degeneration index and Western blot were from 1-3 mice per condition, as indicated. Each data point represents an average value from two fields of view from two individual SCGs cultured in the same dish (a-b) or an individual mouse where both SCGs were cultured for 12 days in the presence of ASOs for 11DIV (c). Data are presented as mean ± SEM. Scale bar represents 50 µm.

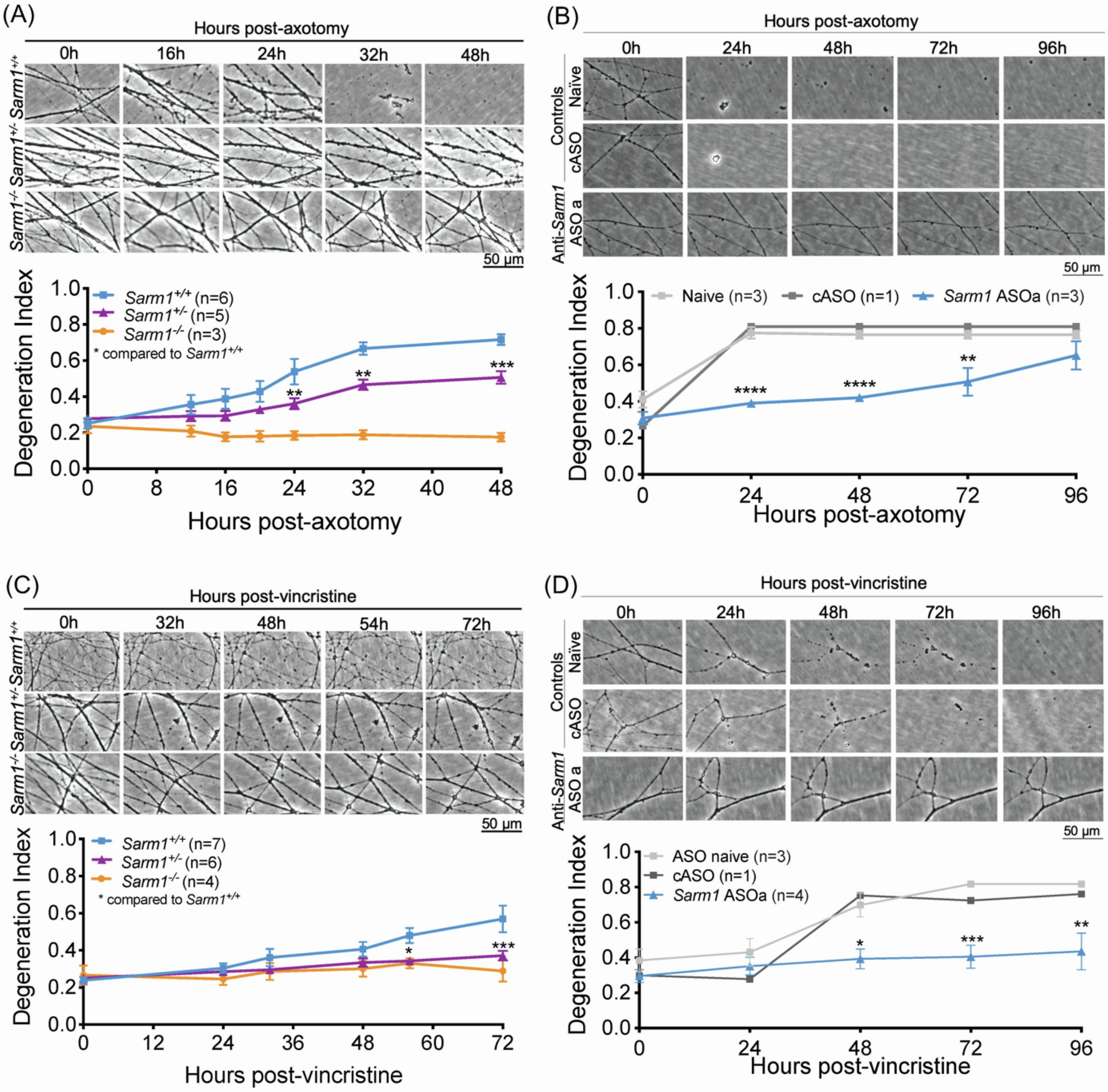
(A)
Hours post-axotomy
0h 16h 24h 32h 48h
Sarm1+/+
Sarm1+/-
Sarm1-/-
50 μm
Degeneration Index
Sarm1+/+ (n=6)
Sarm1+/- (n=5)
Sarm1-/- (n=3)
* compared to Sarm1+/+
Hours post-axotomy
(B)
Hours post-axotomy
0h 24h 48h 72h 96h
Controls
Naïve
cASO
Anti-Sarm1 ASO a
50 μm
Degeneration Index
Naive (n=3) cASO (n=1) Sarm1 ASOa (n=3)
Hours post-axotomy
(C)
Hours post-vincristine
0h 32h 48h 54h 72h
Sarm1+/+
Sarm1+/-
Sarm1-/-
50 μm
Degeneration Index
Sarm1+/+ (n=7)
Sarm1+/- (n=6)
Sarm1-/- (n=4)
* compared to Sarm1+/+
Hours post-vincristine
(D)
Hours post-vincristine
0h 24h 48h 72h 96h
Controls
Naïve
cASO
Anti-Sarm1 ASO a
50 μm
Degeneration Index
ASO naive (n=3)
cASO (n=1)
Sarm1 ASOa (n=4)
Hours post-vincristine

Supplemental Figure 3: *Sarm1* haploinsufficiency or antisense oligonucleotides also delay programmed axon degeneration in DRGs. Confirmation of SCG phenotypes seen in Figures 3 and 5 using DRGs. *Sarm1* haploinsufficient, wild-type, or homozygous null DRGs were cultured for 7DIV and then received either axotomy (a) or 20 nM vincristine (c) and the same fields of view were repeatedly imaged at 20x magnification in phase contrast across the indicated time-course. DRGs receiving plain media or ASOa were cultured for 7DIV with ASOs. At 7DIV, cultures received either axotomy (b) or 20 nM vincristine (d) and the same fields of view were repeatedly imaged at 20x magnification in phase contrast across the indicated time-course. Representative micrographs for each genotype or ASO-treatment group at each timepoint are shown above quantification of axon degeneration. A two-way repeated measures ANOVA was performed followed by Bonferroni post-hoc analysis to determine whether there was a difference between *Sarm1* haploinsufficient and wild-type or homozygous null DRGs (a and c) or Dunnett's post-hoc analysis to determine whether there was a difference or Naive and *Sarm1* ASOa-treated DRGs (b and d). *$p<0.05$; **$p<0.01$; ***$p<0.001$; ****$p<0.0001$. Sample sizes are indicated on graphs for each condition. Each data point represents an average value from three fields of view from three individual DRGs cultured in the same dish. Data are presented as mean $\pm$ SEM. Scale bars represent 50 µm.
