# Supplementary material for: Sarm1 haploinsufficiency or low expression levels after antisense oligonucleotides delay programmed axon degeneration
Source: Cell Rep. 2021 Dec 14;37(11):110108. doi: 10.1016/j.celrep.2021.110108 (PMC8692746; doi:10.1016/j.celrep.2021.110108)
Supplement: Document S2. Article plus supplemental information [file mmc2.pdf]

Cell Reports

Article

Sarm1 haploinsufficiency or low expression levels after antisense oligonucleotides delay programmed axon degeneration

Graphical abstract

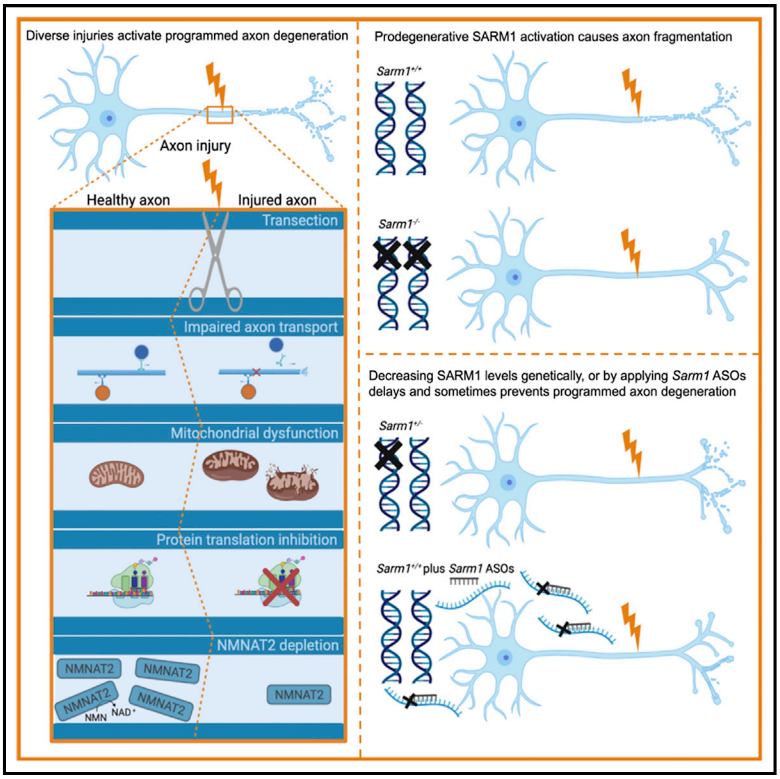

Highlights

- SARM1-dependent axon degeneration occurs after diverse neurotoxic triggers
- Silencing one allele of pro-degenerative SARM1 slows programmed axon degeneration
- *Sarm1* ASOs can mimic this, delaying axon degeneration in multiple contexts
- Decreasing SARM1 expression even partially may be therapeutically valuable

Authors

Stacey Anne Gould, Jonathan Gilley, Karen Ling, Paymaan Jafar-Nejad, Frank Rigo, Michael Coleman

Correspondence

mc469@cam.ac.uk

In brief

By lowering but not completely removing the pro-degenerative protein SARM1, Gould et al. delay axon degeneration in models relevant to pain disorders, mitochondrial dysfunction, impaired protein synthesis, and NMNAT2 disruption. Using antisense oligonucleotides, they demonstrate neuroprotection without total elimination of SARM1, reinforcing the value of SARM1 as a viable therapeutic approach.

Gould et al., 2021, Cell Reports *37*, 110108
December 14, 2021 © 2021 The Authors.
https://doi.org/10.1016/j.celrep.2021.110108

Cell Reports

CellPress OPEN ACCESS

Article

Sarm1 haploinsufficiency or low expression levels after antisense oligonucleotides delay programmed axon degeneration

Stacey Anne Gould,[1] Jonathan Gilley,[1] Karen Ling,[2] Paymaan Jafar-Nejad,[2] Frank Rigo,[2] and Michael Coleman[1,3,4,5,*]

[1]Department of Clinical Neurosciences, University of Cambridge, John van Geest Centre for Brain Repair, Cambridge CB2 0PY, UK

[2]Neuroscience Drug Discovery, Ionis Pharmaceuticals, Inc., 2855 Gazelle Court, Carlsbad, CA 92010, USA

[3]Signalling Programme, The Babraham Institute, Babraham Research Campus, Cambridge CB22 3AT, UK

[4]Twitter: @Lab_Coleman

[5]Lead contact

*Correspondence: mc469@cam.ac.uk

https://doi.org/10.1016/j.celrep.2021.110108

SUMMARY

Activation of the pro-degenerative protein SARM1 after diverse physical and disease-relevant injuries causes programmed axon degeneration. Original studies indicate that substantially decreased SARM1 levels are required for neuroprotection. However, we demonstrate, in *Sarm1* haploinsufficient mice, that lowering SARM1 levels by 50% delays programmed axon degeneration *in vivo* after sciatic nerve transection and partially prevents neurite outgrowth defects in mice lacking the pro-survival factor NMNAT2. *In vitro*, the rate of degeneration in response to traumatic, neurotoxic, and genetic triggers of SARM1 activation is also slowed. Finally, we demonstrate that *Sarm1* antisense oligonucleotides decrease SARM1 levels by more than 50% *in vitro*, which delays or prevents programmed axon degeneration. Combining *Sarm1* haploinsufficiency with antisense oligonucleotides further decreases SARM1 levels and prolongs protection after neurotoxic injury. These data demonstrate that axon protection occurs in a *Sarm1* gene dose-responsive manner and that SARM1-lowering agents have therapeutic potential, making *Sarm1*-targeting antisense oligonucleotides a promising therapeutic strategy.

INTRODUCTION

Sterile alpha and TIR motif containing 1 (SARM1) is a pro-degenerative NADase whose genetic removal confers strong axonal protection after injury and in preclinical disease models (Essuman et al., 2018, 2017; Geisler et al., 2019a; Gerdts et al., 2013; Henninger et al., 2016; Osterloh et al., 2012; White et al., 2019). Removal of *Sarm1* completely prevents the axon growth failure that occurs in mice lacking pro-survival factor nicotinamide mononucleotide adenylyltransferase 2 (NMNAT2), essential for axon growth and maintenance (Gilley et al., 2017, 2015; Gilley and Coleman, 2010). Current models suggest that NMNAT2 loss leads to accumulation of nicotinamide mononucleotide (NMN) (Di Stefano et al., 2017), which directly activates SARM1 NADase activity (Zhao et al., 2019), leading to axon fragmentation.

Recent reports suggest that aberrant activation of programmed axon degeneration can cause human disease. Biallelic loss-of-function (LoF) *NMNAT2* mutations are associated with rare, early-onset, or developmental neuropathies, including polyneuropathy (Huppke et al., 2019) and fetal akinesia deformation sequence (FADS) stillbirth (Lukacs et al., 2019). Phenotypes in the stillborn fetuses resemble the neurogenic muscle deficits in mice lacking *Nmnat2* but appear even more extreme (Gilley et al., 2015, 2013; Hicks et al., 2012). Large variation in *NMNAT2* mRNA levels in human post-mortem brain has also been reported (Ali et al., 2016). Because low expression of *Nmnat2* in mice leads to age-related axon vulnerability and accelerated programmed axon degeneration in response to physical and neurotoxic injury (Gilley et al., 2019), low NMNAT2 expression may contribute to axon loss in common human diseases through aberrant activation of SARM1-dependent programmed axon degeneration. Genome-wide association studies (GWASs) and the role of the SARM1-negative regulator Stathmin-2 (SCG10) (Shin et al., 2012) in amyotrophic lateral sclerosis (ALS) also implicate programmed axon degeneration in human disease (Fogh et al., 2014; Klim et al., 2019; Melamed et al., 2019; van Rheenen et al., 2016). Many animal model studies, including recent data for an ALS/ fronto-temporal degeneration (FTD) mouse (White et al., 2019), suggest that removing *Sarm1* and blocking programmed axon degeneration is a promising therapeutic strategy for axonopathies (Coleman and Höke, 2020; Conforti et al., 2014; DiAntonio, 2019; Loring and Thompson, 2020).

Prophylactic targeting of programmed axon degeneration has particularly interesting potential in chemotherapy-induced neuropathy, where axons in a subset of cancer survivors degenerate (DiAntonio, 2019). Animal and cell culture studies using vincristine, paclitaxel, bortezomib, and oxaliplatin show protection against pain when the programmed axon degeneration pathway is blocked (Geisler et al., 2016; Turkiew et al., 2017; Geisler et al.,

© 2021 The Authors.
This is an open access article under the CC BY license (http://creativecommons.org/licenses/by/4.0/).

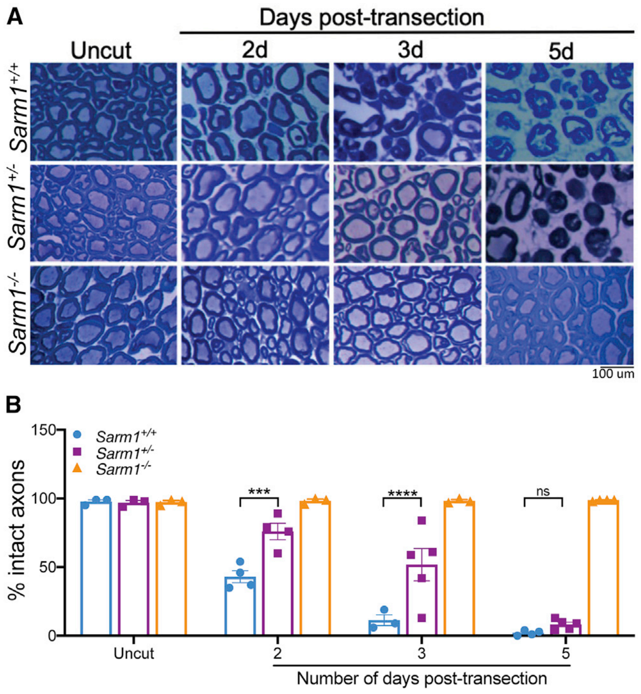

Figure 1. *Sarm1* haploinsufficiency delays the rate of programmed axon degeneration *in vivo* after sciatic nerve transection

(A) Representative images of semi-thin (1 μm) transverse sections through the sciatic nerve of 2- to 3-month-old wild-type, *Sarm1* haploinsufficient, or *Sarm1* homozygous null littermate mice stained with Richardson's solution and imaged using light microscopy at 100× magnification.

(B) Number of intact axons remaining in wild-type, *Sarm1* haploinsufficient, or *Sarm1* homozygous null mice in uncut or cut axons at the indicated number of days after transection. A two-way ANOVA was performed, followed by Dunnett post hoc analysis to determine whether there was a difference between *Sarm1* haploinsufficient and *Sarm1* homozygous wild-type mice at each indicated time point indicated. ***$p < 0.001$, ****$p < 0.0001$; ns, not statistically significant; n = 3–5 per group. Each data point corresponds to quantification of an entire tibial nerve cross-section from a single mouse.

Data are presented as mean ± SEM. The scale bar represents 100 μm.

2019a; Gould et al., 2021). Another potentially responsive cohort could be individuals with rare diseases related to non-lethal variation in genes involved in programmed axon degeneration, such as those experiencing NMNAT2-related polyneuropathy (Huppke et al., 2019), because this activates the pathway very specifically in a way that can be fully rescued in mice (Gilley et al., 2017).

Pharmacological strategies to inhibit or knock down SARM1 are unlikely to completely remove the protein or its activity. Thus, it is important to know whether partial removal is protective. Initial reports indicated that full removal of *Sarm1* in homozygous null mice was needed to protect axons in a transected nerve for 14 days (Osterloh et al., 2012), and *Sarm1* haploinsufficiency did not protect detectably against oxygen-glucose deprivation in hippocampal slice cultures (Kim et al., 2007). However, 70% knockdown of *Sarm1* mRNA protects after axotomy *in vitro*, although the extent to which SARM1 protein was decreased was not reported (Gerdts et al., 2013). The SARM1 haploinsuffiency phenotype is also important for understanding and predicting disease risk in a substantial number of individuals with predicted LoF variants of *SARM1* in the population sequence database gnomAD (Karczewski et al., 2020).

Here we demonstrate that *Sarm1* haploinsufficiency in mice significantly delays programmed axon degeneration after axotomy *in vivo* and in a range of disease and injury models *in vitro*. We also show that a similar or greater decrease in SARM1 levels can be achieved by exogenous application of *Sarm1* antisense oligonucleotides to delay degeneration in primary neuronal cultures by at least as much as in *Sarm1* haploinsufficiency. These results are promising for development of therapies targeting SARM1 in the treatment of relevant human neurological or neurodegenerative disorders involving SARM1 activation.

RESULTS

Sarm1 haploinsufficiency delays programmed axon degeneration *in vivo*

The inability of *Sarm1* haploinsufficiency to protect axons 14 days after sciatic nerve transection (Osterloh et al., 2012) does not preclude protection at earlier time points. Structural signs of degeneration start 32–36 h after lesion in a transected wild-type (*Sarm1*$^{+/+}$) sciatic nerve, with 80% of axons being fragmented by 42 h after lesion and 100% by 48 h (Beirowski et al., 2005). Thus, we assessed axon preservation in *Sarm1* haploinsufficient (*Sarm1*$^{+/-}$) nerves 2–5 days after lesion. We found clear preservation of axons in the largest central (tibial) fascicle of the sciatic nerve from *Sarm1*$^{+/-}$ mice compared with *Sarm1*$^{+/+}$ mice at 2 and 3 days after lesion (Figure 1). Some axons were still preserved 5 days after lesion, but the amount of preservation is not statistically significant compared with *Sarm1*$^{+/+}$ mice and is unlikely to be biologically relevant. This demonstrates that *Sarm1* haploinsufficiency is sufficient to significantly delay programmed axon degeneration after *in vivo* nerve injury and raises the prospect of stronger protective effects in less extreme axon stresses.

Sarm1 haploinsufficiency partially restores neurite outgrowth in embryos lacking functional NMNAT2

Homozygous *Nmnat2* gene trap mice (*Nmnat2*$^{gtE/gtE}$), which express no detectable NMNAT2, die perinatally with nerve growth failure and a hunched posture (Gilley et al., 2013). We generated *Nmnat2*$^{gtE/gtE}$*Sarm1*$^{+/-}$ mice and, although these also died at birth, we did observe *in vivo* rescue of nerve outgrowth relative to *Nmnat2*$^{gtE/gtE}$;*Sarm1*$^{+/+}$ in embryonic day 18 (E18) embryos. First, 1,1'-Dioctadecyl-3,3,3',3'-Tetramethylindocarbocyanine Perchlorate (DiI) labeling of intercostal nerve axons extended as far as in wild-type (*Nmnat2*$^{+/+}$; *Sarm1*$^{+/-}$) or fully rescued double-homozygous null (*Nmnat2*$^{gtE/gtE}$;*Sarm1*$^{-/-}$) intercostals (Gilley et al., 2017; Figure 2A–2C). This contrasts with the severe axon truncation in *Nmnat2*$^{gtE/gtE}$;*Sarm1*$^{+/+}$ embryos, where labeling barely extends beyond the spinal column (Figure 2A). Second, we confirmed previous reports that phrenic nerves fail to innervate diaphragms in *Nmnat2*$^{gtE/gtE}$;*Sarm1*$^{+/+}$ embryos (Di Stefano et al., 2017; Gilley et al., 2015, 2013) but found evidence of phrenic nerves reaching the lateral diaphragm (LD) of *Nmnat2*$^{gtE/gtE}$;*Sarm1*$^{+/-}$ mice by immunostaining, even when the extent of innervation is relatively limited compared with *Nmnat2*$^{gtE/gtE}$;*Sarm1*$^{-/-}$ diaphragm, which reaches the costal diaphragm (CD; Figure 2D).

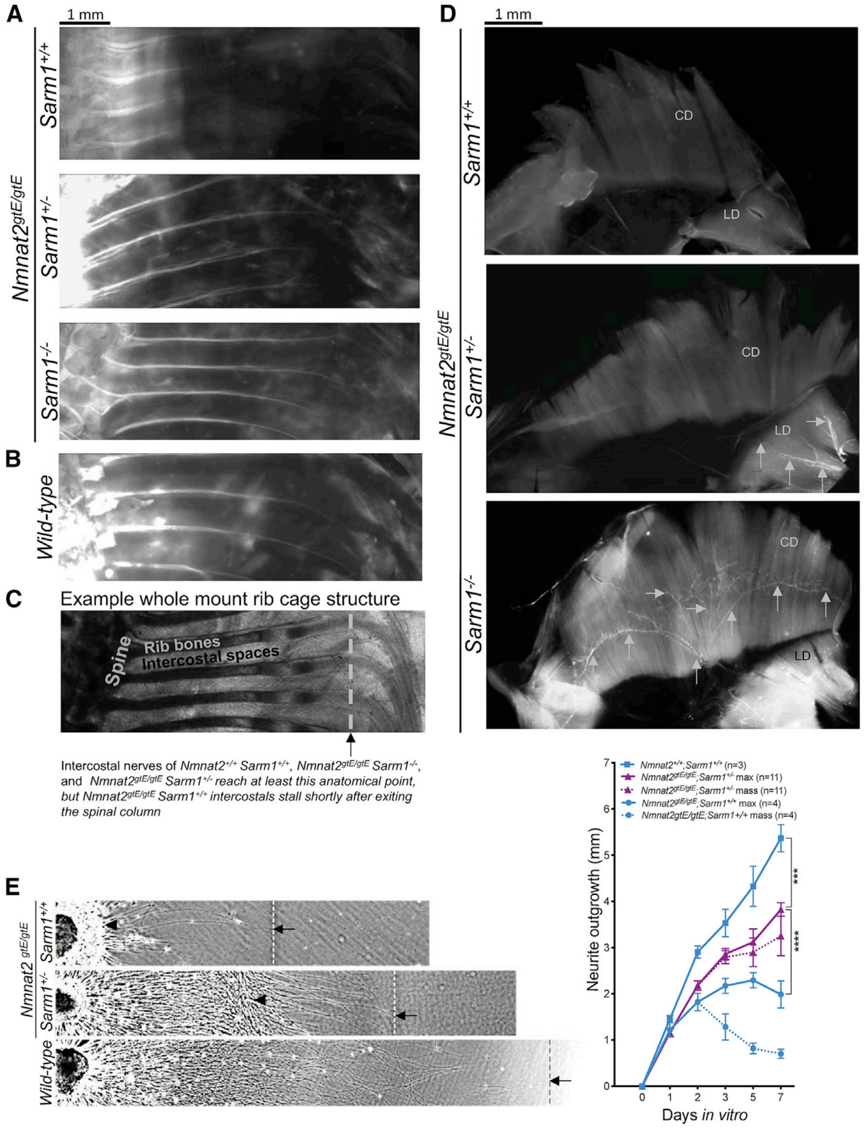

Figure 2. *Sarm1* haploinsufficiency partially restores neurite outgrowth deficits in mice lacking NMNAT2

(A and B) DiI labeling of intercostal nerves from *Nmnat2*$^{gtE/gtE}$;*Sarm1*$^{+/+}$, *Nmnat2*$^{gtE/gtE}$;*Sarm1*$^{+/-}$, *Nmnat2*$^{gtE/gtE}$;*Sarm1*$^{-/-}$ (A), and wild-type (B) E18.5 embryos.

(C) An example of the whole-mount rib cage structure; the gray line and black arrow indicate the point where intercostal nerves from *Nmnat2*$^{gtE/gtE}$;*Sarm1*$^{+/-}$, *Nmnat2*$^{gtE/gtE}$;*Sarm1*$^{-/-}$, and wild-type embryos extend beyond but which those from *Nmnat2*$^{gtE/gtE}$;*Sarm1*$^{+/+}$ embryos fail to reach.

(D) βIII-Tubulin staining (indicated by gray arrows) of a whole-mount diaphragm shows the extent of innervation by the phrenic nerve in *Nmnat2*$^{gtE/gtE}$;*Sarm1*$^{+/+}$, *Nmnat2*$^{gtE/gtE}$;*Sarm1*$^{+/-}$, and *Nmnat2*$^{gtE/gtE}$;*Sarm1*$^{-/-}$ E18.5 embryos at 5× magnification. As reported previously (Gilley et al., 2013), there was no detectable fluorescent signal showing innervation in the *Nmnat2*$^{gtE/gtE}$;*Sarm1*$^{+/+}$ diaphragm. LD, lateral diaphragm; CD, costal diaphragm.

(E) Finally, neurite outgrowth was quantified in DRGs from E13.5 embryos across 7 days *in vitro*. Each data point represents average neurite length from three fields of view from three DRG explants cultured together from one embryo. The triangle indicates the point to which the majority of neurites grow, referred to as “mass,” and the arrow and dotted line indicate the point to which the longest neurites grow, referred to as “max.” Where only “max” is indicated, “mass” is the same. A two-way repeated-measures ANOVA was performed, followed by Tukey post hoc analysis to determine whether there was a difference between *Sarm1* haploinsufficient and *Sarm1* homozygous wild-type DRGs lacking NMNAT2 or DRGs wild type for both genes. ****$p < 0.0001$. E18.5 embryos for the *in vivo* studies were obtained from multiple *Nmnat2*$^{+/gtE}$;*Sarm1*$^{+/-}$ × *Nmnat2*$^{+/gtE}$;*Sarm1*$^{+/-}$ crosses, which also yielded many embryos of non-desired genotypes. Therefore, E13.5 embryos for the *in vitro* outgrowth were obtained from a mixture of *Nmnat2*$^{+/gtE}$;*Sarm1*$^{+/+}$ × *Nmnat2*$^{+/gtE}$;*Sarm1*$^{+/+}$ and *Nmnat2*$^{gtE/gtE}$;*Sarm1*$^{-/-}$ x *Nmnat2*$^{gtE/gtE}$;*Sarm1*$^{-/-}$ crosses to ensure that all genotypes needed were present in litters at the time of dissection and plating. Data are presented as mean ± SEM. Scale bars represent 1 mm.

These data suggest partial reversal of neurite outgrowth deficits, albeit insufficient to prevent respiratory failure. Quantifying nerve outgrowth *in vivo* has a number of caveats, so we also quantified axon outgrowth in cultured dorsal root ganglia (DRGs). We found a statistically significant, partial reversal of neurite outgrowth deficits in DRG explants from *Nmnat2*$^{gtE/gtE}$ mice haploinsufficient for *Sarm1* compared with those wild type for *Sarm1*, which are severely truncated (Figure 2E).

Sarm1 haploinsufficiency delays programmed axon degeneration triggered by a variety of physical and toxic insults

To rapidly explore the ability of *Sarm1* haploinsufficiency to protect against a range of Wallerian and Wallerian-like pathologies, we switched to superior cervical ganglion (SCG) cultures, which enabled us to test multiple initiators of programmed axon degeneration. After confirming that SARM1 protein levels were decreased by around 50% in *Sarm1* haploinsufficient SCGs (Figure 3A), axotomy was performed to confirm that the level of intermediate protection seen *in vivo* was also present in culture, allowing other mechanisms of axonal stress to be explored. Indeed, there was a significant delay in degeneration, particularly notable 8 h after cut in *Sarm1* haploinsufficient SCGs compared with those from littermate wild types (Figure 3B). Axon degeneration in *Sarm1* haploinsufficient SCGs was also delayed up to 54 h when induced by vincristine (a chemotherapeutic agent that blocks axonal transport and causes degeneration of distal axons) (Figure 3C)

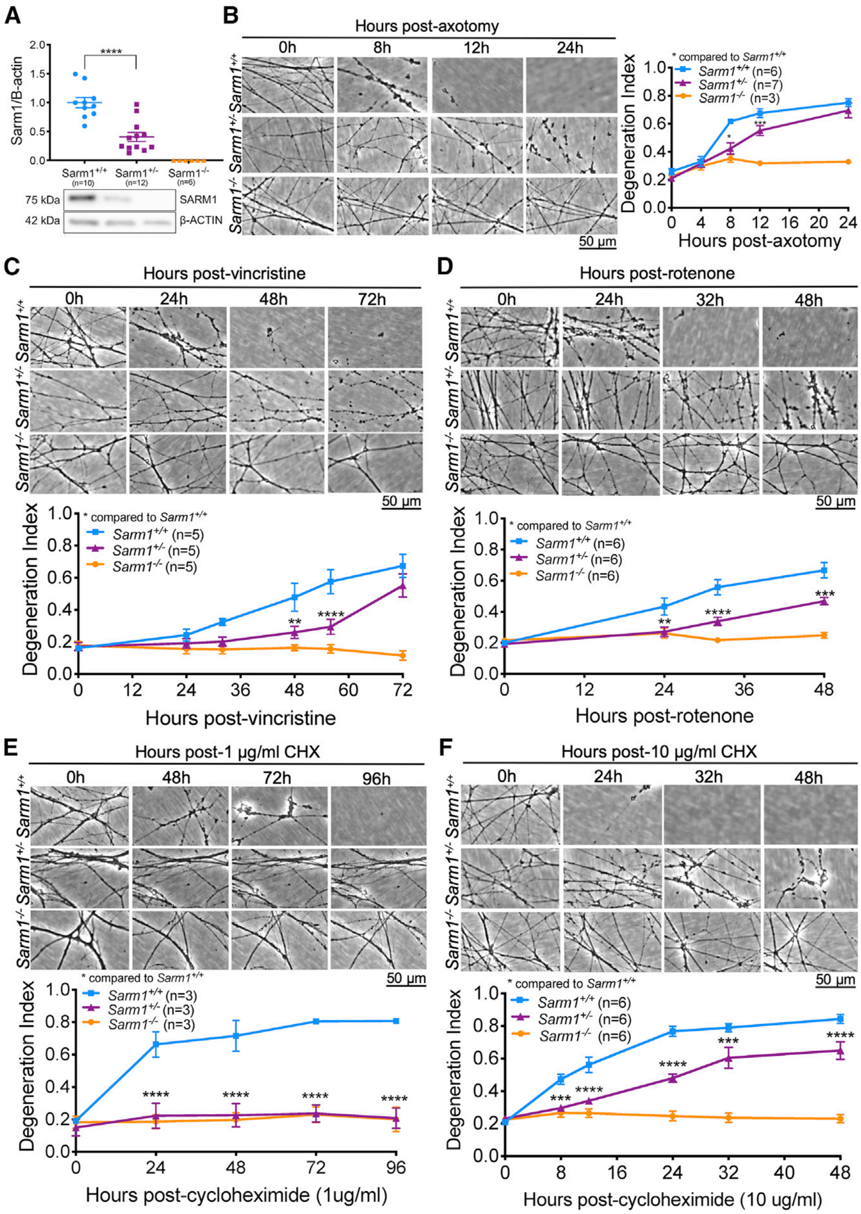

Figure 3. *Sarm1* haploinsufficiency lowers SARM1 levels and confers protection against diverse triggers of programmed axon degeneration

(A) Western blot shows that SARM1 levels are lower in *Sarm1* haploinsufficient SCGs cultured for 7 DIV than *Sarm1* homozygous wild-type SCGs when normalized to β-actin; representative bands are shown below the quantification. Each data point corresponds to an individual mouse where both SCGs were cultured in the same dish for 7 days and then collected for western blotting.

(B–F) At 7 DIV, cultured SCGs received a trigger for programmed axon degeneration, and the same fields of view were imaged repeatedly at 20× magnification in phase contrast across the indicated time course. These triggers were axotomy (B), 20 nM vincristine (C), 25 μM rotenone (D), 1 μg/mL CHX (E), or 10 μg/mL CHX (F), which have all been shown previously to induce programmed axon degeneration. Representative micrographs for each genotype at each time point are shown next to or above quantification of axon degeneration. A one-way ANOVA was performed, followed by an unpaired t test to determine whether there was a significant difference between *Sarm1* haploinsufficient and *Sarm1* homozygous wild-type SCG SARM1 band intensity. A two-way repeated-measures ANOVA was performed, followed by Bonferroni post hoc analysis to determine whether there was a difference between *Sarm1* haploinsufficient and *Sarm1* homozygous wild-type SCGs. $^*p < 0.05$, $^{**}p < 0.01$, $^{***}p < 0.001$, $^{****}p < 0.0001$. Sample sizes are indicated on graphs for each condition; at least three experimental replicates were run for each experiment on separate occasions. Each data point represents an average value from two fields of view from two individual SCGs cultured in the same dish.

Data are presented as mean ± SEM. Scale bars represent 50 μm. See Figure S3 for the effects of *Sarm1* haploinsufficiency on the rate of degeneration in injured DRGs.

and up to 48 h when induced by rotenone (which inhibits complex I, leading to mitochondrial dysfunction and generation of reactive oxygen species [ROS]) (Figure 3D).

We then modeled protein translation inhibition through cycloheximide (CHX) administration. Labile proteins, such as the axon survival factor NMNAT2 (Gilley and Coleman, 2010) and SCG10 (Shin et al., 2012; Walker et al., 2017), are depleted rapidly after CHX treatment and can initiate programmed axon degeneration (Gilley and Coleman, 2010). We demonstrate here that complete removal of *Sarm1* prevents degeneration at a low (1 μg/mL) and high dose (10 μg/mL) of CHX for the duration of the experiment (Figures 3E and 3F). Notably, at the lower dose, *Sarm1* haploinsufficiency was found to be as protective as complete removal of *Sarm1*, with neurites remaining intact up to 96 h (Figure 3E). After 96 h, low-dose CHX caused detachment of *Sarm1*$^{-/-}$ and *Sarm1*$^{+/-}$ neurites independent of degeneration. At a higher dose of CHX, *Sarm1* haploinsufficiency conferred partial protection up to 48 h, after which the neurites detached (Figure 3F).

SARM1 levels can be decreased by exogenous application of antisense oligonucleotides, and this delays programmed axon degeneration *in vitro*

Next we tested whether SARM1 protein levels could be decreased by antisense oligonucleotides (ASOs) and whether this had a protective effect. One control ASO (cASO) and two *Sarm1* ASOs (ASOa and ASOb) were used. Application of *Sarm1* ASOa to DRG explants decreased SARM1 levels to around 3% of control levels (Figure 4A). This completely reversed the neurite outgrowth deficit in *Nmnat2*$^{gtE/gtE}$ DRGs with outgrowth comparable with that of *Nmnat2*$^{+/+}$ DRGs treated

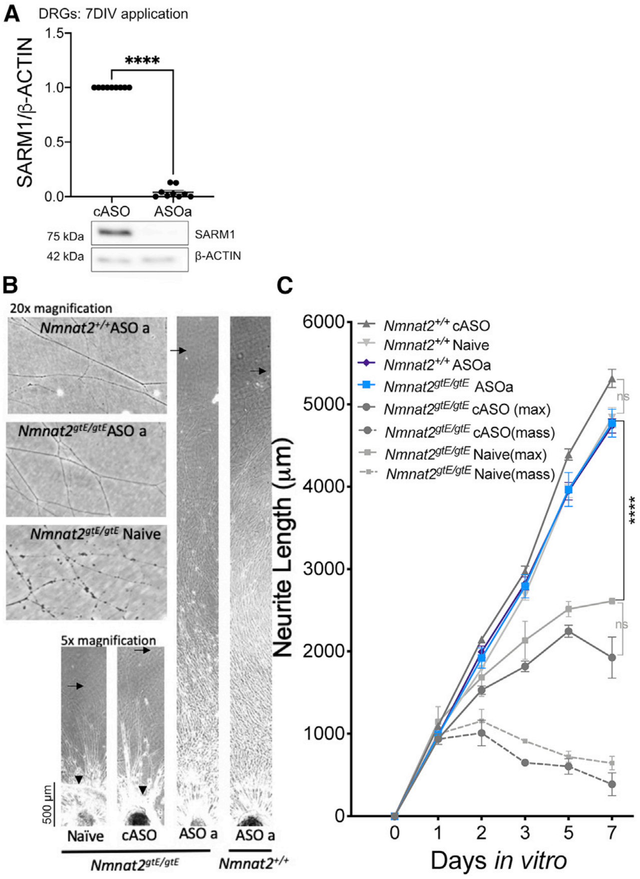

Figure 4. *Sarm1* ASOs completely reverse the neurite outgrowth deficit in DRGs lacking NMNAT2

(A) Western blot shows that SARM1 levels are lower in *Sarm1* ASO-treated DRGs cultured for 7 DIV than cASO-treated DRGs when normalized to β-actin; representative bands are shown below the quantification. Each data point corresponds to an individual mouse where three DRGs were cultured for 7 days in the presence of ASOs.

(B) Representative phase-contrast images of full-length and distal neurites (at 5× and 20× magnification, respectively) of DRGs from E13.5 embryos.

(C) Quantification of neurite outgrowth of *Nmnat2*$^{+/+}$*Sarm1*$^{+/+}$ in the presence of plain medium (naive), cASO, or ASOa across 7 days *in vitro*. The triangle indicates the point to which the majority of neurites grow, referred to as "mass," and arrows indicate the point to which the longest neurites grow, referred to as "max." Where only "max" is indicated, "mass" is the same. The scale bar represents 50 μm. A two-way ANOVA was performed, followed by Tukey post hoc analysis to determine whether there were significant differences between the groups indicated on the graph. An unpaired t test was used to determine whether there was a significant difference between the cASO- and ASOa-treated DRG explant SARM1 band intensity. ****$p < 0.0001$. Sample sizes for outgrowth were from 4–6 embryos per condition, with each experimental condition being run on three separate occasions. Each data point represents average neurite length from three fields of view from three DRGs cultured together from one mouse.

Data are presented as mean ± SEM. See Figure S1 for the effects of *Sarm1* ASOs on neurite outgrowth and SARM1 protein levels in *Sarm1*$^{+/+}$ SCGs.

with or without the cASO (Figures 4B and 4C). Even the most distal ends of the neurites, which, in *Nmnat2*$^{gtE/gtE}$ cASO explants, show signs of degeneration, were intact in *Nmnat2*$^{gtE/gtE}$ DRGs receiving ASOa (Figure 4A; 20× magnification panel). SCGs treated with any of the ASOs or co-application of both for 6 days exhibited normal outgrowth (Figures S1A and S1B) and SARM1 levels were decreased significantly to around 17%–25% of control levels (Figure S1C). Importantly, and as expected, this decreased SARM1 expression conferred delayed degeneration against axotomy, vincristine, and CHX application (Figures 5A–5D).

Neurites of ASO-treated cells remained intact and healthy for up to 12 days, the longest duration of the *in vitro* assays performed (Figures S2A and S2B), and SARM1 levels were not decreased further after prolonged application (Figure S2C). We also found that the capacity for *Sarm1* haploinsufficiency or ASO application to delay programmed axon degeneration is transferrable from SCGs to DRGs in the axotomy and vincristine models (Figure S3).

Further decreasing SARM1 levels by applying *Sarm1* ASOs to *Sarm1* haploinsufficient SCGs prolongs protection against vincristine-induced degeneration *in vitro*

Finally, to assess whether further lowering, but not eliminating, SARM1 levels could further delay the rate of axon degeneration, we assessed whether application of *Sarm1* ASOs could prolong the already delayed rate of degeneration in *Sarm1* haploinsufficient SCGs. This paradigm also enabled us to simulate application of SARM1-targeting drugs to individuals who are genetically haploinsufficient, as predicted from sequences within the gnomAD human population sequence database (Karczewski et al., 2020). Unsurprisingly, SARM1 levels are decreased significantly further in *Sarm1* haploinsufficient SCGs after ASOa or ASOa+b application (Figure 6A) to 9% or 7% of wild-type levels, respectively. There is also a trend toward decreased expression after ASOb application (to 14%). Nevertheless, this decrease is biologically relevant because it corresponds to a delay in axon degeneration up to 96 h after vincristine compared with control-treated *Sarm1* haploinsufficient SCGs, which completely degenerate by 72 h (Figure 6B).

DISCUSSION

Here we demonstrate that removing one *Sarm1* allele, leading to an approximately 50% decrease in SARM1 protein levels, is sufficient to delay programmed axon (Wallerian) degeneration, despite previous reports (Kim et al., 2007; Osterloh et al., 2012). This applies to highly damaging *in vivo* models of sciatic nerve transection (for up to 3 days) and NMNAT2-related developmental defects as well as to a range of *in vitro* non-transection injuries. Importantly, we demonstrate that it is possible to decrease SARM1 protein levels via exogenous application of ASOs *in vitro* to levels even lower than those present in *Sarm1* haploinsufficient neurons and that this is protective in a battery of *in vitro* assays that trigger programmed axon degeneration. The rate of degeneration *in vitro* can be delayed further in *Sarm1* haploinsufficient SCGs that receive *Sarm1* ASOs, where SARM1 levels are decreased by around 95% compared with wild-type controls. These demonstrations are encouraging for continued work toward anti-SARM1 therapies for use in human disease and add another therapeutic option

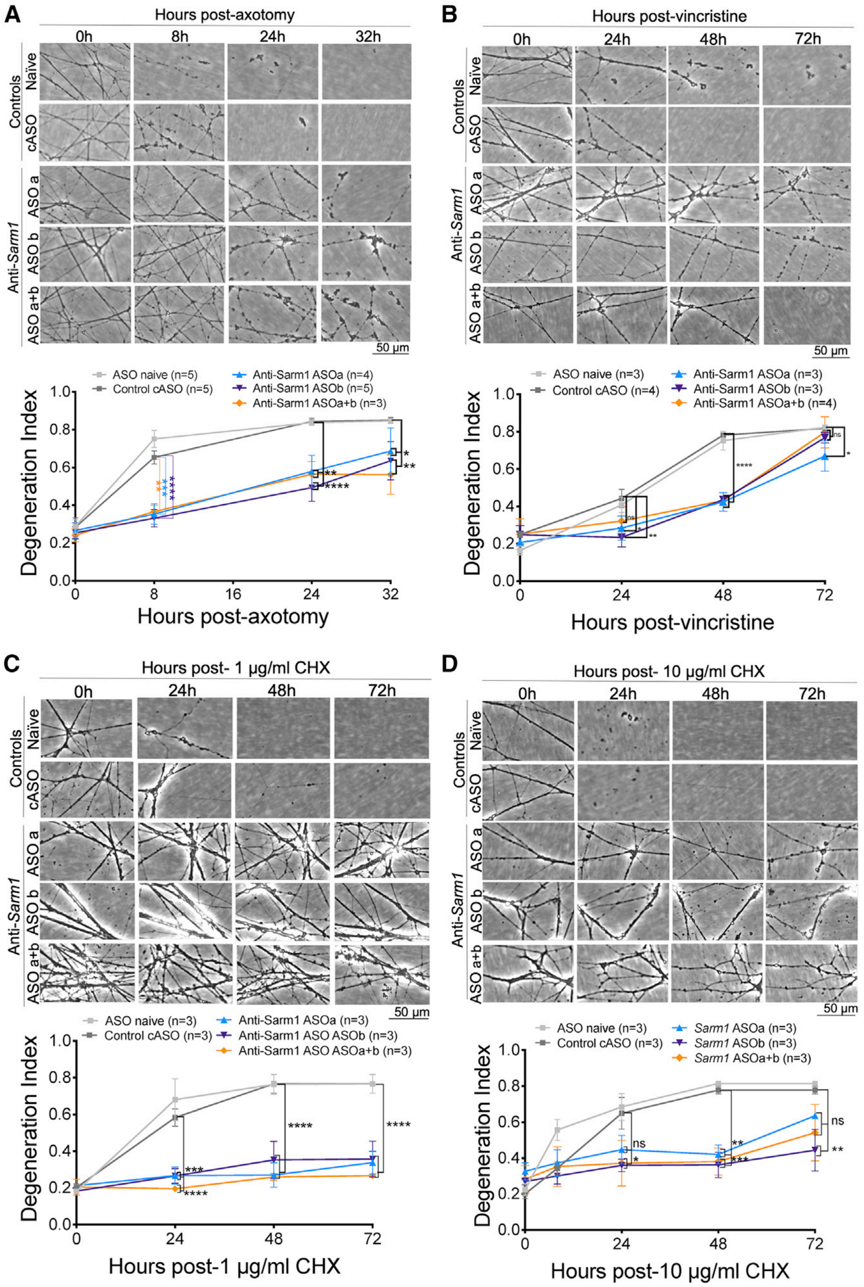

Figure 5. *Sarm1* ASOs confer protection against diverse triggers of programmed axon degeneration

(A–D) Cultured SCGs received ASOs for 6 DIV (starting from the day after being plated in plain culture medium). At 7 DIV, cultured SCGs received a trigger for programmed axon degeneration, and the same fields of view were imaged repeatedly at 20× magnification in phase contrast across the indicated time course. These triggers were axotomy (A), 20 nM vincristine (B), 1 μg/mL CHX (C), or 10 μg/mL CHX (D). Representative micrographs for each genotype at each time point are shown above quantification of axon degeneration. A two-way repeated-measures ANOVA was performed, followed by Bonferroni post hoc analysis to determine whether there was a difference between cASO and *Sarm1* ASOs (ASOa, ASOb, or combined ASOa+ASOb) for the experiments in (B)–(D). For the experiment in (A), mixed-effects analysis was used to account for a single missing value from each of the *Sarm1* ASO-treated conditions at the final time point (because of neurite detachment preventing the final value being obtained). *$p < 0.05$, **$p < 0.01$, ***$p < 0.001$, ****$p < 0.0001$. Sample sizes are indicated on the graphs for each condition; at least three replicates were run for each experiment on separate occasions. Each data point represents an average value from two fields of view from two individual SCGs cultured in the same dish. Data are presented as mean ± SEM. Scale bars represent 50 μm. See Figures S1 and S2 for additional control experiments in ASO-treated SCGs and Figure S3 for the effects of *Sarm1* ASOs on the rate of degeneration in injured DRGs.

to SARM1-based gene therapy (Geisler et al., 2019b) and small-molecule inhibition of SARM1-TIR domain NADase activity (Hughes et al., 2021; Loring et al., 2020) currently being explored.

The level of protection observed in *Sarm1* haploinsufficient mice after sciatic nerve transection, where 75% axons remain intact 2 days and 50% 3 days after transection, exceeds that of complete removal of CRMP4 or dual leucine zipper-bearing kinase (DLK) (Girouard et al., 2020; Miller et al., 2011) and is comparable with calpastatin overexpression in the optic nerve 3 days after lesion (Yang et al., 2013). Because partial removal of protein activity is more achievable clinically than complete removal, the strength of protection seen with partial lowering of SARM1 levels in comparison with complete removal of other proteins makes it an attractive target.

A second extreme *in vivo* model used was mice lacking NMNAT2, which die at birth (Gilley et al., 2013; Hicks et al., 2012) but survive and live normal healthy lifespans when *Sarm1* is removed (Gilley et al., 2017, 2015). The ability of *Sarm1* haploinsufficiency to partially rescue in this model further confirms the partial protective phenotype of *Sarm1* haploinsufficiency *in vivo*, confirming that *Sarm1* haploinsufficiency can modulate axon health in the context of altered NMNAT2 levels. Our finding that *Sarm1* haploinsufficiency slows the rate of programmed axon degeneration *in vitro* in response to diverse triggers (chemotherapeutic vincristine, mitochondrial dysfunction induced by rotenone, and protein translation inhibition by low- and high-dose CHX) also suggests that human conditions involving mitochondrial deficits and protein synthesis impairments may be alleviated by anti-SARM1 therapies. Therefore, we tested whether it was possible to lower SARM1 levels and achieve a delay in degeneration through exogenous application of ASOs. Indeed, we

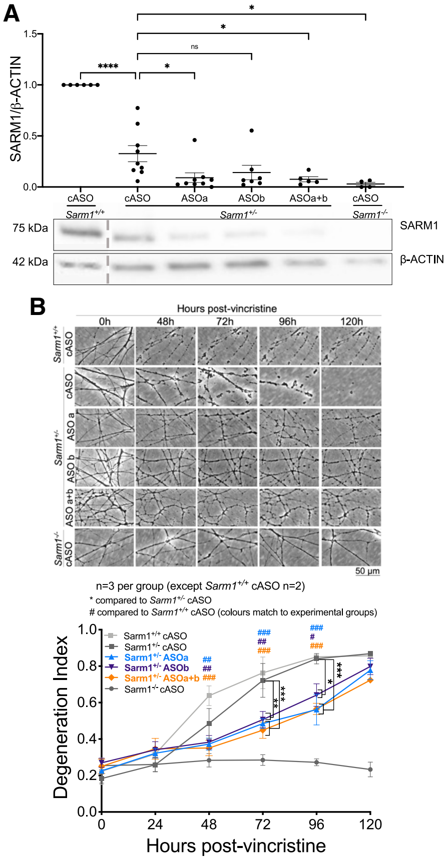

Figure 6. Combining *Sarm1* haploinsufficiency and ASOs further lowers SARM1 levels and further delays axon degeneration

(A) Western blot showing that SARM1 levels are lower in *Sarm1* haploinsufficient SCGs treated with *Sarm1* ASOs than in *Sarm1* haploinsufficient SCGs treated with cASO when normalized to β-actin; representative bands are shown below the quantification; a dotted gray line indicates that the sample was in a non-adjacent well on the same blot.

(B) At 7 DIV, cultured SCGs received 20 nM vincristine, which triggers programmed axon degeneration, and the same fields of view were imaged repeatedly at 20× magnification in phase contrast across the indicated time course. Representative micrographs for each genotype and treatment group at each time point are shown above quantification of axon degeneration. For western blot quantification, a one-way ANOVA was performed, followed by Dunnett's post hoc analysis to determine whether there was a significant difference between *Sarm1* haploinsufficient cASO-treated and *Sarm1* haploinsufficient *Sarm1* ASO-treated SCGs. For quantification of axon degeneration, a two-way repeated-measures ANOVA was performed, followed by Dunnett's post hoc analysis to determine whether there was a difference between cASO- and *Sarm1* ASO-treated *Sarm1* haploinsufficient SCGs.

*$p < 0.05$, **$p < 0.01$, ***$p < 0.001$, ****$p < 0.0001$. Sample sizes are indicated on graphs for each condition; at least three replicates were run for each experiment on separate occasions. Each data point represents an individual mouse where both SCGs were cultured for 7 days in the presence of ASOs for 6 DIV and then collected for western blotting (A) or an average value from two fields of view from two individual SCGs cultured in the same dish (B and C). Data are presented as mean ± SEM. The scale bar represents 50 μm.

demonstrate that this confers an axon-protective phenotype across diverse triggering events to an equal or greater extent than *Sarm1* haploinsufficiency.

The most striking effect noted with application of the ASOs was seen in neurite outgrowth of DRGs lacking NMNAT2. Unlike in *Sarm1* haploinsufficient DRGs, which only partially rescued DRG neurite outgrowth, knockdown with ASOs completely rescued outgrowth, with even the most distal ends of axons appearing as healthy as those with wild-type NMNAT2 levels. The strength of this rescue is likely due to more efficient knockdown of *Sarm1* observed in DRGs compared with SCGs (by 95% and 80%, respectively), although this may reflect subtle differences in the timing of application rather than intrinsic differences between these neuron types.

We demonstrated that lowering levels of SARM1 through removal of one allele or application of ASOs can protect against axon degeneration in response to protein translation inhibition by low-dose CHX as efficiently as complete removal of SARM1. Furthermore, the extent to which axon fragmentation is delayed after high-dose CHX is greater after ASO application (Figure 5C) than in *Sarm1* haploinsufficient neurons (Figure 3E), with degeneration being prevented for 72–96 h, equaling the protective effects of complete *Sarm1* removal. These data suggest that less complete SARM1 knockdown can confer strong protection in diseases affecting protein translation. We administered CHX to model slow, chronic depletion of proteins to mimic protein aggregation or translation disorders. Suppression of the initiation and elongation phases of translation (mediated by eukaryotic initiation or the elongation factors eIFs and eEFs, respectively), caused by protein aggregation, is seen in neurodegenerative diseases (Freeman and Mallucci, 2016; Knight et al., 2020). This results in chronic depletion of proteins that are required for creating and maintaining healthy axons and synapses. Indeed, inhibition of phosphorylated eIF2α-mediated protein translation suppression alleviates readouts of neurodegeneration in prion-infected mice and an FTD model of tauopathy (Halliday et al., 2017). Removal of *Sarm1* or the presence of Wallerian degeneration slow (WLD^S) (or other overexpressed NMNATs, which negatively regulate programmed axon degeneration) show promise in preventing disease progression in models of FTD (Ali et al., 2012; Ljungberg et al., 2012; White et al., 2019). Together with the results presented here, this suggests that targeting SARM1 when protein translation is inhibited could be beneficial, in addition to prophylactic administration in predictable neurotoxic injuries, such as chemotherapy, or in rare human diseases that involve loss of or altered NMNAT2 function, such as rare polyneuropathies (Huppke et al., 2019). Further work in animal models is required to confirm this.

The gnomAD database indicates that there are humans with at least one *SARM1* LoF allele (Karczewski et al., 2020), and we have identified additional functionally confirmed missense LoF alleles (M. Ademi et al., 2021, Peripheral Nerve Soc., conference), bringing the combined prevalence of SARM1 partial LoF to around at least 0.4%. This may be subject to variation between populations and could be even higher because of noncoding *LoF* variants yet to be studied. The SARM1 locus has been associated with ALS (Fogh et al., 2014; van Rheenen et al., 2016), with gain-of-function (GoF) mutations found in the human ALS population altering axon susceptibility to usually subdegenerative insults (Gilley et al., 2021).

Our data suggest that individuals with already lowered SARM1 expression may experience stronger neuroprotective effects in response to SARM1-lowering therapies compared with humans without LoF alleles. We speculate that individuals with *Sarm1* LoF alleles may respond strongly to treatments targeting SARM1 because they may reach levels close to those of homozygous null mice. However, this needs to be confirmed *in vivo*. Alternatively, in individuals with *NMNAT2* LoF or *SARM1* GoF linked to human disease, SARM1-targeting therapies may normalize the vulnerability of affected axons and prevent or delay the onset or progression of disease. Further study is required *in vivo* to confirm these findings in relevant disease models. However, the results we present here suggest that targeting SARM1 is a promising strategy.

Conclusions

Here we demonstrate that programmed axon degeneration can be delayed by lowering SARM1 levels and not only by completely removing the protein. This can be achieved by genetic removal of one allele as well as through exogenous application of ASOs. Our data suggest that some disorders, like those caused by loss of NMNAT2 or involving defective protein translation (as modeled by CHX) or axonal transport deficits (as modeled by vincristine), are more likely to respond to SARM1-modifying therapies than others, such as physical transection injuries. Therefore, selection of the most appropriate diseases as well as populations of affected individuals will be crucial for demonstrating clinical efficacy of anti-SARM1 therapies. Further studies need to explore whether the effects reported in this study can be transferred to *in vivo* disease models and to establish the functional effects of predicted human *Sarm1* GoF and LoF mutations on axon health and vulnerability.

Limitations of the study

This study strongly supports the notion that lowering SARM1 levels delays axon degeneration after nerve transection and lowering NMNAT2 *in vivo* and in multiple models with and without physical neurite injury *in vitro*. However, the practicalities of lowering SARM1 levels *in vivo* using ASOs need to be empirically determined. In addition, although peripheral nerve transection is arguably the most stringent of axon degeneration models, the effect on each disease-relevant model, including those without physical injury, would need to be determined case by case to determine the full therapeutic value of SARM1-lowering strategies in chronic disease states. Finally, this study focused on the effects of lowering SARM1 in the nervous system, so exploration of any potential effects in non-nervous tissue could also be assessed in future work.

STAR★METHODS

Detailed methods are provided in the online version of this paper and include the following:

- KEY RESOURCES TABLE
- RESOURCE AVAILABILITY
  - Lead contact
  - Materials availability
  - Data and code availability
- EXPERIMENTAL MODEL AND SUBJECT DETAILS
  - Animals
  - Primary cell cultures
- METHOD DETAILS
  - Sciatic nerve transection surgeries
  - Sciatic nerve dissections, processing, imaging, and quantification
  - DiI staining of intercostal nerves
  - Diaphragm dissection and staining
  - Primary cell culture assays of Wallerian and Wallerian-like degeneration
  - Antisense oligonucleotides
  - Neurite outgrowth
  - Western blotting
- QUANTIFICATION AND STATISTICAL ANALYSIS

SUPPLEMENTAL INFORMATION

Supplemental information can be found online at https://doi.org/10.1016/j.celrep.2021.110108.

ACKNOWLEDGMENTS

We would like to thank Robert Adalbert and Andrea Loreto for providing useful training. Studies of the *Sarm1* haploinsufficiency phenotype in isolation were funded by a BBSRC CASE studentship co-sponsored by Takeda (Cambridge) Ltd. Subsequent work on ASOs was funded by the van Geest Foundation, Funds for Women Graduates, The University of Cambridge Lundgren Award, and Wolfson College.

AUTHOR CONTRIBUTIONS

S.A.G. performed all experiments and data analyses. S.A.G. designed experiments and prepared the manuscript with input from J.G. and M.C. The antisense oligonucleotides were provided by K.L., P.J.-N., and F.R. along with advice on dosing. All authors read and approved the manuscript.

DECLARATION OF INTERESTS

K.L., P.J.-N., and F.R. are employees of Ionis Pharmaceuticals, Inc., and part of this study was jointly funded by Takeda (Cambridge) Ltd.

INCLUSION AND DIVERSITY

We worked to ensure sex balance in the selection of non-human subjects. One or more of the authors of this paper self-identifies as living with a disability. One or more of the authors of this paper received support from a program designed to increase minority representation in science. While citing references scientifically relevant for this work, we also actively worked to promote gender balance in our reference list.

Received: March 4, 2021
Revised: August 24, 2021
Accepted: November 17, 2021
Published: December 14, 2021

REFERENCES

Ali, Y.O., Allen, H.M., Yu, L., Li-Kroeger, D., Bakhshizadehmahmoudi, D., Hatcher, A., McCabe, C., Xu, J., Bjorklund, N., and Taglialatela, G. (2016). NMNAT2:HSP90 complex mediates proteostasis in proteinopathies. PLoS Biol. *14* (6), e1002472.. https://doi.org/10.1371/journal.pbio.1002472.

Ali, Y.O., Ruan, K., and Zhai, R.G. (2012). NMNAT suppresses tau-induced neurodegeneration by promoting clearance of hyperphosphorylated tau oligomers in a Drosophila model of tauopathy. Hum. Mol. Genet. *21*, 237–250.

Beirowski, B., Berek, L., Adalbert, R., Wagner, D., Grumme, D.S., Addicks, K., Ribchester, R.R., and Coleman, M.P. (2004). Quantitative and qualitative analysis of Wallerian degeneration using restricted axonal labelling in YFP-H mice. J. Neurosci. Methods *134*, 23–35.

Beirowski, B., Adalbert, R., Wagner, D., Grumme, D.S., Addicks, K., Ribchester, R.R., and Coleman, M.P. (2005). The progressive nature of Wallerian degeneration in wild-type and slow Wallerian degeneration (WldS) nerves. BMC Neurosci. *6*, 6.

Buckmaster, E.A., Perry, V.H., and Brown, M.C. (1995). The rate of Wallerian degeneration in cultured neurons from wild type and C57BL/WldS mice depends on time in culture and may be extended in the presence of elevated K+ levels. Eur. J. Neurosci. *7*, 1596–1602.

Chen, C.-Y., Lin, C.-W., Chang, C.-Y., Jiang, S.-T., and Hsueh, Y.-P. (2011). Sarm1, a negative regulator of innate immunity, interacts with syndecan-2 and regulates neuronal morphology. J. Cell Biol. *193*, 769–784.

Coleman, M.P., and Höke, A. (2020). Programmed axon degeneration: from mouse to mechanism to medicine. Nat. Rev. Neurosci. *21*, 183–196.

Conforti, L., Gilley, J., and Coleman, M.P. (2014). Wallerian degeneration: an emerging axon death pathway linking injury and disease. Nat. Rev. Neurosci. *15*, 394–409.

Di Stefano, M., Loreto, A., Orsomando, G., Mori, V., Zamporlini, F., Hulse, R.P., Webster, J., Donaldson, L.F., Gering, M., Raffaelli, N., et al. (2017). NMN Deamidase Delays Wallerian Degeneration and Rescues Axonal Defects Caused by NMNAT2 Deficiency In Vivo. Curr. Biol. *27*, 784–794.

DiAntonio, A. (2019). Axon degeneration: mechanistic insights lead to therapeutic opportunities for the prevention and treatment of peripheral neuropathy. Pain *160* (*Suppl 1*), S17–S22.

Essuman, K., Summers, D.W., Sasaki, Y., Mao, X., DiAntonio, A., and Milbrandt, J. (2017). The SARM1 Toll/Interleukin-1 Receptor Domain Possesses Intrinsic NAD^+ Cleavage Activity that Promotes Pathological Axonal Degeneration. Neuron *93*, 1334–1343.e5.

Essuman, K., Summers, D.W., Sasaki, Y., Mao, X., Yim, A.K.Y., DiAntonio, A., and Milbrandt, J. (2018). TIR Domain Proteins Are an Ancient Family of NAD^+-Consuming Enzymes. Curr. Biol. *28*, 421–430.e4.

Fogh, I., Ratti, A., Gellera, C., Lin, K., Tiloca, C., Moskvina, V., Corrado, L., Sorarù, G., Cereda, C., Corti, S., et al.; SLAGEN Consortium and Collaborators (2014). A genome-wide association meta-analysis identifies a novel locus at 17q11.2 associated with sporadic amyotrophic lateral sclerosis. Hum. Mol. Genet. *23*, 2220–2231.

Freeman, O.J., and Mallucci, G.R. (2016). The UPR and synaptic dysfunction in neurodegeneration. Brain Res. *1648* (*Pt B*), 530–537.

Geisler, S., Doan, R.A., Strickland, A., Huang, X., Milbrandt, J., and DiAntonio, A. (2016). Prevention of vincristine-induced peripheral neuropathy by genetic deletion of SARM1 in mice. Brain *139*, 3092–3108.

Geisler, S., Doan, R.A., Cheng, G.C., Cetinkaya-Fisgin, A., Huang, S.X., Höke, A., Milbrandt, J., and DiAntonio, A. (2019a). Vincristine and bortezomib use distinct upstream mechanisms to activate a common SARM1-dependent axon degeneration program. JCI Insight *4*, 129920.

Geisler, S., Huang, S.X., Strickland, A., Doan, R.A., Summers, D.W., Mao, X., Park, J., DiAntonio, A., and Milbrandt, J. (2019b). Gene therapy targeting SARM1 blocks pathological axon degeneration in mice. J. Exp. Med. *216*, 294–303.

Gerdts, J., Summers, D.W., Sasaki, Y., DiAntonio, A., and Milbrandt, J. (2013). Sarm1-mediated axon degeneration requires both SAM and TIR interactions. J. Neurosci. *33*, 13569–13580.

Gilley, J., and Coleman, M.P. (2010). Endogenous Nmnat2 is an essential survival factor for maintenance of healthy axons. PLoS Biol. *8*, e1000300.

Gilley, J., Adalbert, R., Yu, G., and Coleman, M.P. (2013). Rescue of peripheral and CNS axon defects in mice lacking NMNAT2. J. Neurosci. *33*, 13410–13424.

Gilley, J., Orsomando, G., Nascimento-Ferreira, I., and Coleman, M.P. (2015). Absence of SARM1 rescues development and survival of NMNAT2-deficient axons. Cell Rep. *10*, 1974–1981.

Gilley, J., Ribchester, R.R., and Coleman, M.P. (2017). Sarm1 Deletion, but Not Wld^S, Confers Lifelong Rescue in a Mouse Model of Severe Axonopathy. Cell Rep. *21*, 10–16.

Gilley, J., Mayer, P.R., Yu, G., and Coleman, M.P. (2019). Low levels of NMNAT2 compromise axon development and survival. Hum. Mol. Genet. *28*, 448–458.

Gilley, J., Jackson, O., Pipis, M., Estiar, M.A., Gan-Or, Z., Goutman, S.A., Harms, M.B., Kaye, J., Lima, L., Genomics, Q.S., et al. (2021). Enrichment of SARM1 alleles encoding variants with constitutively hyperactive NADase in patients with ALS and other motor nerve disorders. medRxiv. https://doi.org/10.1101/2021.06.17.21258268.

Girouard, M.-P., Simas, T., Hua, L., Morquette, B., Khazaei, M.R., Unsain, N., Johnstone, A.D., Rambaldi, I., Sanz, R.L., Di Raddo, M.E., et al. (2020). Collapsin Response Mediator Protein 4 (CRMP4) Facilitates Wallerian Degeneration and Axon Regeneration following Sciatic Nerve Injury. eNeuro *7*, ENEURO.0479-19.2020.

Gould, S.A., White, M., Wilbrey, A.L., Pór, E., Coleman, M.P., and Adalbert, R. (2021). Protection against oxaliplatin-induced mechanical and thermal hypersensitivity in $Sarm1^{-/-}$ mice. Exp. Neurol. *338*, 113607.

Halliday, M., Radford, H., Zents, K.A.M., Molloy, C., Moreno, J.A., Verity, N.C., Smith, E., Ortori, C.A., Barrett, D.A., Bushell, M., and Mallucci, G.R. (2017). Repurposed drugs targeting eIF2α-P-mediated translational repression prevent neurodegeneration in mice. Brain *140*, 1768–1783.

Henninger, N., Bouley, J., Sikoglu, E.M., An, J., Moore, C.M., King, J.A., Bowser, R., Freeman, M.R., and Brown, R.H., Jr. (2016). Attenuated traumatic axonal injury and improved functional outcome after traumatic brain injury in mice lacking Sarm1. Brain *139*, 1094–1105.

Hicks, A.N., Lorenzetti, D., Gilley, J., Lu, B., Andersson, K.-E., Miligan, C., Overbeek, P.A., Oppenheim, R., and Bishop, C.E. (2012). Nicotinamide mononucleotide adenylyltransferase 2 (Nmnat2) regulates axon integrity in the mouse embryo. PLoS ONE *7*, e47869.

Hughes, R.O., Bosanac, T., Mao, X., Engber, T.M., DiAntonio, A., Milbrandt, J., Devraj, R., and Krauss, R. (2021). Small Molecule SARM1 Inhibitors Recapitulate the $SARM1^{-/-}$ Phenotype and Allow Recovery of a Metastable Pool of Axons Fated to Degenerate. Cell Rep. *34*, 108588.

Huppke, P., Wegener, E., Gilley, J., Angeletti, C., Kurth, I., Drenth, J.P.H., Stadelmann, C., Barrantes-Freer, A., Brück, W., Thiele, H., et al. (2019). Homozygous NMNAT2 mutation in sisters with polyneuropathy and erythromelalgia. Exp. Neurol. *320*, 112958.

Karczewski, K.J., Francioli, L.C., Tiao, G., Cummings, B.B., Alföldi, J., Wang, Q., Collins, R.L., Laricchia, K.M., Ganna, A., Birnbaum, D.P., et al.; Genome Aggregation Database Consortium (2020). The mutational constraint spectrum quantified from variation in 141,456 humans. Nature *581*, 434–443.

Kim, Y., Zhou, P., Qian, L., Chuang, J.-Z., Lee, J., Li, C., Iadecola, C., Nathan, C., and Ding, A. (2007). MyD88-5 links mitochondria, microtubules, and JNK3 in neurons and regulates neuronal survival. J. Exp. Med. *204*, 2063–2074.

Klim, J.R., Williams, L.A., Limone, F., Guerra San Juan, I., Davis-Dusenbery, B.N., Mordes, D.A., Burberry, A., Steinbaugh, M.J., Gamage, K.K., Kirchner, R., et al. (2019). ALS-implicated protein TDP-43 sustains levels of STMN2, a mediator of motor neuron growth and repair. Nat. Neurosci. *22*, 167–179.

Knight, J.R.P., Garland, G., Pöyry, T., Mead, E., Vlahov, N., Sfakianos, A., Grosso, S., De-Lima-Hedayioglu, F., Mallucci, G.R., von der Haar, T., et al. (2020). Control of translation elongation in health and disease. Dis. Model. Mech. *13*, dmm043208.

Ljungberg, M.C., Ali, Y.O., Zhu, J., Wu, C.-S., Oka, K., Zhai, R.G., and Lu, H.-C. (2012). CREB-activity and nmnat2 transcription are down-regulated prior to neurodegeneration, while NMNAT2 over-expression is neuroprotective, in a mouse model of human tauopathy. Hum. Mol. Genet. *21*, 251–267.

Loreto, A., and Gilley, J. (2020). Axon Degeneration Assays in Superior Cervical Ganglion Explant Cultures. Methods Mol. Biol. *2143*, 15–24.

Loring, H.S., and Thompson, P.R. (2020). Emergence of SARM1 as a Potential Therapeutic Target for Wallerian-type Diseases. Cell Chem. Biol. *27*, 1–13.

Loring, H.S., Parelkar, S.S., Mondal, S., and Thompson, P.R. (2020). Identification of the first noncompetitive SARM1 inhibitors. Bioorg. Med. Chem. *28*, 115644.

Lukacs, M., Gilley, J., Zhu, Y., Orsomando, G., Angeletti, C., Liu, J., Yang, X., Park, J., Hopkin, R.J., Coleman, M.P., et al. (2019). Severe biallelic loss-of-function mutations in nicotinamide mononucleotide adenylyltransferase 2 (NMNAT2) in two fetuses with fetal akinesia deformation sequence. Exp. Neurol. *320*, 112961.

Melamed, Z., López-Erauskin, J., Baughn, M.W., Zhang, O., Drenner, K., Sun, Y., Freyermuth, F., McMahon, M.A., Beccari, M.S., Artates, J.W., et al. (2019). Premature polyadenylation-mediated loss of stathmin-2 is a hallmark of TDP-43-dependent neurodegeneration. Nat. Neurosci. *22*, 180–190.

Miller, B., Press, C., Daniels, R.W., Sasaki, Y., Milbrandt, J., and DiAntonio, A. (2011). A DLK and JNK Dependent Axon Self-destruction Program Promotes Wallerian Degeneration. Nat. Neurosci. *12*, 387–389.

Osterloh, J.M., Yang, J., Rooney, T.M., Fox, A.N., Adalbert, R., Powell, E.H., Sheehan, A.E., Avery, M.A., Hackett, R., Logan, M.A., et al. (2012). dSarm/Sarm1 is required for activation of an injury-induced axon death pathway. Science *337*, 481–484.

Sasaki, Y., Vohra, B.P.S., Lund, F.E., and Milbrandt, J. (2009). Nicotinamide mononucleotide adenylyl transferase-mediated axonal protection requires enzymatic activity but not increased levels of neuronal nicotinamide adenine dinucleotide. J. Neurosci. *29*, 5525–5535.

Shin, J.E., Miller, B.R., Babetto, E., Cho, Y., Sasaki, Y., Qayum, S., Russler, E.V., Cavalli, V., Milbrandt, J., and DiAntonio, A. (2012). SCG10 is a JNK target in the axonal degeneration pathway. Proc. Natl. Acad. Sci. USA *109*, E3696–E3705.

Summers, D.W., DiAntonio, A., and Milbrandt, J. (2014). Mitochondrial dysfunction induces Sarm1-dependent cell death in sensory neurons. J. Neurosci. *34*, 9338–9350.

Swayze, E.E., Siwkowski, A.M., Wancewicz, E.V., Migawa, M.T., Wyrzykiewicz, T.K., Hung, G., Monia, B.P., and Bennett, C.F. (2007). Antisense oligonucleotides containing locked nucleic acid improve potency but cause significant hepatotoxicity in animals. Nucleic Acids Res. *35*, 687–700.

Turkiew, E., Falconer, D., Reed, N., and Höke, A. (2017). Deletion of Sarm1 gene is neuroprotective in two models of peripheral neuropathy. J. Peripher. Nerv. Syst. *22*, 162–171.

van Rheenen, W., Shatunov, A., Dekker, A.M., McLaughlin, R.L., Diekstra, F.P., Pulit, S.L., van der Spek, R.A.A., Võsa, U., de Jong, S., Robinson, M.R., et al. (2016). Genome-wide association analyses identify new risk variants and the genetic architecture of amyotrophic lateral sclerosis. Nat. Genet. *48* (*9*), 1043–1048. https://doi.org/10.1038/ng.3622.

Walker, L.J., Summers, D.W., Sasaki, Y., Brace, E.J., Milbrandt, J., and DiAntonio, A. (2017). MAPK signaling promotes axonal degeneration by speeding the turnover of the axonal maintenance factor NMNAT2. eLife *6*, e22540.

Wang, M.-S., Wu, Y., Culver, D.G., and Glass, J.D. (2000). Pathogenesis of axonal degeneration: parallels between Wallerian degeneration and vincristine neuropathy. J. Neuropathol. Exp. Neurol. *59*, 599–606.

White, M.A., Lin, Z., Kim, E., Henstridge, C.M., Pena Altamira, E., Hunt, C.K., Burchill, E., Callaghan, I., Loreto, A., Brown-Wright, H., et al. (2019). Sarm1 deletion suppresses TDP-43-linked motor neuron degeneration and cortical spine loss. Acta Neuropathol. Commun. *7*, 166.

Yang, J., Weimer, R.M., Kallop, D., Olsen, O., Wu, Z., Renier, N., Uryu, K., and Tessier-Lavigne, M. (2013). Regulation of axon degeneration after injury and in development by the endogenous calpain inhibitor calpastatin. Neuron *80*, 1175–1189.

Zhao, Z.Y., Xie, X.J., Li, W.H., Liu, J., Chen, Z., Zhang, B., Li, T., Li, S.L., Lu, J.G., Zhang, L., et al. (2019). A Cell-Permeant Mimetic of NMN Activates SARM1 to Produce Cyclic ADP-Ribose and Induce Non-apoptotic Cell Death. iScience *15*, 452–466.

CellPress
OPEN ACCESS

STAR★METHODS

KEY RESOURCES TABLE

REAGENT or RESOURCE	SOURCE	IDENTIFIER
Antibodies		
SARM1	Previously published	Chen et al., 2011
β-ACTIN	Sigma-Aldrich	Cat#A5316; RRID: AB_476697
Goat anti-mouse IgG (H+L)-HRP Conjugate	Bio-Rad	Cat#1721011; RRID:AB_2617113
Chemicals, peptides, and recombinant proteins		
Vincristine	Sigma-Aldrich	CAS#2068-78-2
Rotenone	Sigma-Aldrich	CAS#83-79-4
Cycloheximide	Sigma-Aldrich	CAS#66-81-9
Experimental models: Organisms/strains		
Sarm1$^{-/-}$ mice	Previously published	Kim et al., 2007
Nmnat2$^{+/gtE}$ mice	Previously published	Gilley et al., 2013
Oligonucleotides		
ASOa: 5′-GGCAACCTCAC CTTACTCAA-3′	This paper	N/A
ASOb: 5′-GTACCAGGTAG TTACAGAGC-3′	This paper	N/A
cASO: 5′-CCTATAGGAC TATCCAGGAA-3	This paper	N/A
Software and algorithms		
Fiji	Open-source image analysis software	https://doi.org/10.1038/nmeth.2019
Degeneration Index plugin	Adapted from previous publication	Sasaki et al., 2009

RESOURCE AVAILABILITY

Lead contact

Further information and requests for resources and reagents should be directed to and will be fulfilled by the Lead Contact, Michael Coleman (mc469@cam.ac.uk).

Materials availability

The antisense oligonucleotides used in this study can be available from Ionis Pharmaceuticals. The lead contact can be approached for more details on obtaining the antisense oligonucleotides, and the specific oligonucleotide sequences are listed in the Key resources table.

Data and code availability

Data can be made available upon request to the Lead Contact, Michael Coleman (mc469@cam.ac.uk). No code was used in this study. Any additional information required to reanalyze the data reported in this paper is available from the Lead Contact upon request.

EXPERIMENTAL MODEL AND SUBJECT DETAILS

Animals

All animal work was performed in accordance with the Animal Scientific Procedures Act (ASPA), 1986 and Home Office regulations under project license numbers 70/7620 and P98A03BF9. All animals were kept on a 12:12 hour light:dark cycle at a constant temperature of 19°C in a pathogen-free environment with *ad libitum* access to drinking water and standard rodent chow (unless otherwise stated). Both male and female mice were used for

in vivo experiments (at 2-3 months of age) and *in vitro* experiments (perinatally, at embryonic day 13.5-18.5, or postnatally at day 0-2, as defined in each experiment).

Sarm1$^{+/-}$ mice were generated by crossing C57BL/6Babr wild-type mice with *Sarm1* (*MyD88-5*) homozygous null mice (Kim et al., 2007) with littermates then being crossed to generate mixed genotype litters. Littermates were used for all *in vivo* and *in vitro* experiments assessing the protective effect of *Sarm1* haploinsufficiency. The *in vitro* studies assessing the effects of antisense oligonucleotides in *Sarm1* haploinsufficient cultures were done using mice generated from either Babraham C57BL/6Babr mice, the *Sarm1* homozygous null line (Kim et al., 2007), or crosses between the two lines. Mice for the *in vitro* outgrowth data were generated from two separate timed-matings, with wild-type controls (*Nmnat2*$^{+/+}$;*Sarm1*$^{+/+}$) and mice lacking NMNAT2 wild-type for *Sarm1* (*Nmnat2*$^{gtE/gtE}$;*Sarm1*$^{+/+}$) being obtained from a cross between two *Nmnat2*$^{+/gtE}$;*Sarm1*$^{+/+}$ mice (Gilley et al., 2013). Mice lacking NMNAT2 also haploinsufficient for *Sarm1* (*Nmnat2*$^{gtE/gtE}$;*Sarm1*$^{+/-}$) were obtained from a cross between *Nmnat2*$^{+/gtE}$;*Sarm1*$^{+/+}$ and *Nmnat2*$^{gtE/gtE}$;*Sarm1*$^{-/-}$ mice (Gilley et al., 2015). For the *in vivo* study, double hemizygous mice were generated then crossed together to generate littermate mice for the *in vivo* study where neurite outgrowth was assessed in *Nmnat2*$^{gtE/gtE}$ mice with wild-type, haploinsufficient, or homozygous null *Sarm1* status.

Primary cell cultures

Primary cultures were prepared as described (Loreto and Gilley, 2020). Superior cervical ganglia (SCGs) were dissected from P0-P2 pups and dorsal root ganglia (DRGs) from E13.5 embryos. Dissected ganglia were plated on 35 mm culture dishes precoated with poly-L-lysine (20 μg/ml overnight; Sigma) and laminin (20 μg/ml for 1–2 h; Sigma), as previously described (Gilley and Coleman, 2010). Ganglia were plated in culture medium which comprised Dulbecco's Modified Eagle's Medium (DMEM) (type 41966029; ThermoFisher Scientific) supplemented with 2% B27 supplement (type 17504-044; GIBCO) and 1% penicillin-streptomycin (type P4333; Sigma). In addition, 4 μM aphidicolin (Merck) and 50 ng/ml 2.5S NGF (Invitrogen) were added fresh to the culture medium as it was replaced every 2-3 days.

METHOD DETAILS

Sciatic nerve transection surgeries

A slightly modified version of the method published by Osterloh et al., (2012) was employed to surgically transect the right sciatic nerve of adult mice. Briefly, adult mice (aged 2-3 months) were anesthetized with isoflurane, and the skin on their right hind limb was shaved and cleaned with denatured ethanol. Sterile surgical technique was used to make an incision directly beneath the hip joint, and the gluteal muscles were separated carefully with a pair of forceps. The sciatic nerve was transected immediately adjacent to the sciatic notch with a pair of sterile surgical scissors. The gluteal muscles were then placed back into their original anatomical position, and the overlying skin glued closed using Vetbond.

Sciatic nerve dissections, processing, imaging, and quantification

At 2, 3, and 5 days post-lesion, mice were euthanized via cervical dislocation followed by exsanguination and nerve segments 4 mm distal to the lesion up to the point of nerve trifurcation were collected from both the cut and uncut hindlimbs and fixed with 4% paraformaldehyde and 2.5% glutaraldehyde in 0.1 M PBS, pH 7.4 for at least 72h at 4°C. Fixed nerves were then washed in 0.1 M PBS, placed in 1% osmium tetroxide for 2 h, and dehydrated in 50%, 70%, 80%, 90%, 95%, and 100% ethanol, then 100% propylene oxide. Nerves were then embedded in Durcupan resin (Fluka Chemie) and polymerized for 48 h at 60°C, after which transverse semi-thin sections (100 μM) were cut on a Leica ultramicrotome, stained with Richardson's solution, and imaged on a light microscope.

To quantify the number of intact and degenerated axons, a complete cross section of the fascicle supplying the tibial nerve was counted for each mouse, since it has been demonstrated that axons fragment uniformly along the entire length after transection (Beirowski et al., 2004). The criteria for categorizing axons is the same as that previously published by Osterloh et al., (2012). Criteria for intact axons were; the presence of normal myelin sheaths, uniform axoplasm, and the absence of aggregated mitochondria. The experimenter was blinded to mouse genotype for the surgeries, sectioning, imaging, and quantification.

DiI staining of intercostal nerves

E18.5 embryos of the appropriate genotypes were immersion fixed in 4% paraformaldehyde. After at least one week, intercostal nerves of the embryos were labeled using the lipophilic dye DiI (1,l''-dioctadecyll3,3,3,''3''tetramethylindocarbo-cyanine perchlorate), as previously described by Gilley et al., (2013). DiI crystals were inserted into a rostrocaudal incision along the entire spinal cord, then kept at 37°C in 4% paraformaldehyde for 8 weeks, to allow diffusion of the dye through neuronal membranes thereby labeling all nerves exiting the spinal cord. After 8 weeks of labeling, ribcages were dissected and cleaned then immersed in an increasing PBS glycerol series from 25%, 50%, 75%, up to 100% glycerol for optimal clearing.

Diaphragm dissection and staining

Diaphragms were dissected at the same time as ribcages from the same E18.5 embryos stained with DiI. As described by Di Stefano et al., 2017, diaphragms were washed in PBS then permeabilized and blocked in PTX (PBS, 0.5% Triton X-100) with 2% BSA for 2 hours at room temperature and incubated overnight at room temperature with a rabbit polyclonal anti-βIII-tubulin (TUJ1) antibody (Sigma, T2200) in blocking solution (1:500 dilution). After 3x washes in PTX, diaphragms were incubated with an AlexaFluor488-conjugated anti-rabbit antibody (1:200 dilution) in blocking solution for 5 hours at RT. After another 3x washes in PTX, diaphragms were

mounted in Vectashield on glass sides and imaged using an inverted DMi8 Leica fluorescence microscope using 5x and 20x objectives.

Primary cell culture assays of Wallerian and Wallerian-like degeneration

In all of the *in vitro* degeneration assays, the same field of neurites were imaged in phase contrast at the time points indicated using a DMi8 Leica moving stage inverted epifluorescence microscope. In all assays, using both DRGs and SCGs, neurites were allowed to extend from ganglia for 7DIV before the inducer of degeneration (described below) was applied along with a media immediately preceding time point 0 h.

For axotomy, neurites were physically cut with a sterile scalpel (No.22 blade) several mm away from the ganglion, completely separating the cell bodies from the distal neurites (Buckmaster et al., 1995). Stocks (1000x) of vincristine and rotenone in DMSO were diluted 1:1000 in culture medium to final concentrations of 20 nM vincristine and 10 μM rotenone, previously shown to induce degeneration (Summers et al., 2014; Wang et al., 2000). An aqueous stock solution of CHX in DMSO (Sigma) was diluted 1:1000 or 1:100 in culture media to give final 1 μg/ml and 10 μg/ml CHX concentrations, respectively, as previously described (Gilley et al., 2010). The extent to which distal neurites degenerated was quantified using the Degeneration Index plugin for ImageJ (FIJI) we developed based on a previously reported method (Sasaki et al., 2009).

Antisense oligonucleotides

The antisense oligonucleotides (ASOs) were developed and synthesized by Ionis Pharmaceuticals. Synthesis and purification were performed as previously described (Swayze et al., 2007). The ASOs are 20 nucleotides in length, chemically modified MOE-gapmer oligonucleotides, wherein the central gap segment comprising ten 2′-deoxyribonucleotides that are flanked on the 5′ and 3′ wings by five 2′MOE modified nucleotides. Internucleotide linkages are phosphorothioate interspersed with phosphodiester, and all cytosine residues are 5′-methylcytosines. The sequences of the two *Sarm1* targeting ASOs are: ASOa, 5′-GGCAACCTCACCTTACTCAA-3′ ; ASOb, 5′-GTACCAGGTAGTTACAGAGC-3′, and a non-targeting ASO cASO, 5′-CCTATAGGACTATCCAGGAA-3′. ASO stock solution were each formulated at 14 mM in calcium and magnesium free phosphate buffered saline. For all experiments, stocks were diluted 1:3000 in culture medium to a final concentration of 4.6 μM. Where antisense oligonucleotides were combined, the culture medium comprised a 50:50 mix of ASOa and ASOb, resulting in the final culture medium containing 2.3 μM of each ASO. Antisense oligonucleotide-containing medium was applied to cultures for the durations indicated.

Neurite outgrowth

Similar to previously described experiments (Gilley et al., 2013 and 2015), radial neurite outgrowth was imaged in phase-contrast at low magnification (5x objective) using the 'Tile Scan' function of a DMi8 Leica moving stage inverted epifluorescence microscope. This was done at the same time on each specified day after plating. Neurite length on each day was determined by taking the average of two measurements of representative neurite outgrowth for each explant (2-3 ganglia per dish) and generating an average for each dish.

For the antisense oligonucleotide reversal of neurite outgrowth deficit in mice lacking NMNAT2, 3 DRGs from each embryo of the appropriate genotype $Nmnat2^{gtE/gtE}$/$Sarm1^{+/+}$ or $Nmnat2^{+/+}$/$Sarm1^{+/+}$ were plated in 5 separate 35 mm tissue culture dishes and treated with either normal culture medium, or medium containing control cASO, *Sarm1* ASOa, *Sarm1* ASOb, or combined *Sarm1* ASOa+b. Some detachment of cultures from the dish was experienced so those datapoints are excluded (DRG cultures treated with *Sarm1* ASOb or combined *Sarm1* ASOa+b for both genotypes between 3 and 5DIV) but there was no indication of toxicity for these ASOs.

Western blotting

DRGs and SCGs were collected and the pellet resuspended in Laemmli buffer containing 1% 2-mercaptoethanol. Samples were heated to 100°C for 3 mins then vortexed. Samples were separated on 4%–20% Mini-PROTEAN TGX Precast Protein Gels (Bio-Rad) and transferred onto a methanol-activated Immobilon-P PDVF Membrane (MerckMillipore) by wet blotting. TBST containing 5% milk (Sigma) was used to block membranes and add antibodies; primary antibodies (SARM1 [Chen et al., 2011] and β-ACTIN from Sigma Cat#A5316) were applied overnight at 4°C and secondary antibodies (Goat anti-mouse IgG (H+L)-HRP Conjugate from Bio-Rad Cat#1721011) at RT for 2 h before the Pierce ECL Western Blotting Substrate (ThermoFisher) was added 5 minutes prior to chemiluminescence exposure using a UviTech Alliance chemiluminescence machine.

QUANTIFICATION AND STATISTICAL ANALYSIS

Data generated were compiled in Microsoft Excel. Statistical analyses were performed and graphs created using Prism 8 (GraphPad). Data were quantified and analyzed as indicated in each subsection of the Experimental model and subject details. Specific details of samples sizes and the analyses performed can be found in the figures and figure legends, in addition to the aforementioned subsections of the STAR Methods.

Cell Reports, Volume *37*

Supplemental information

Sarm1 haploinsufficiency or low expression levels after antisense oligonucleotides delay programmed axon degeneration

Stacey Anne Gould, Jonathan Gilley, Karen Ling, Paymaan Jafar-Nejad, Frank Rigo, and Michael Coleman

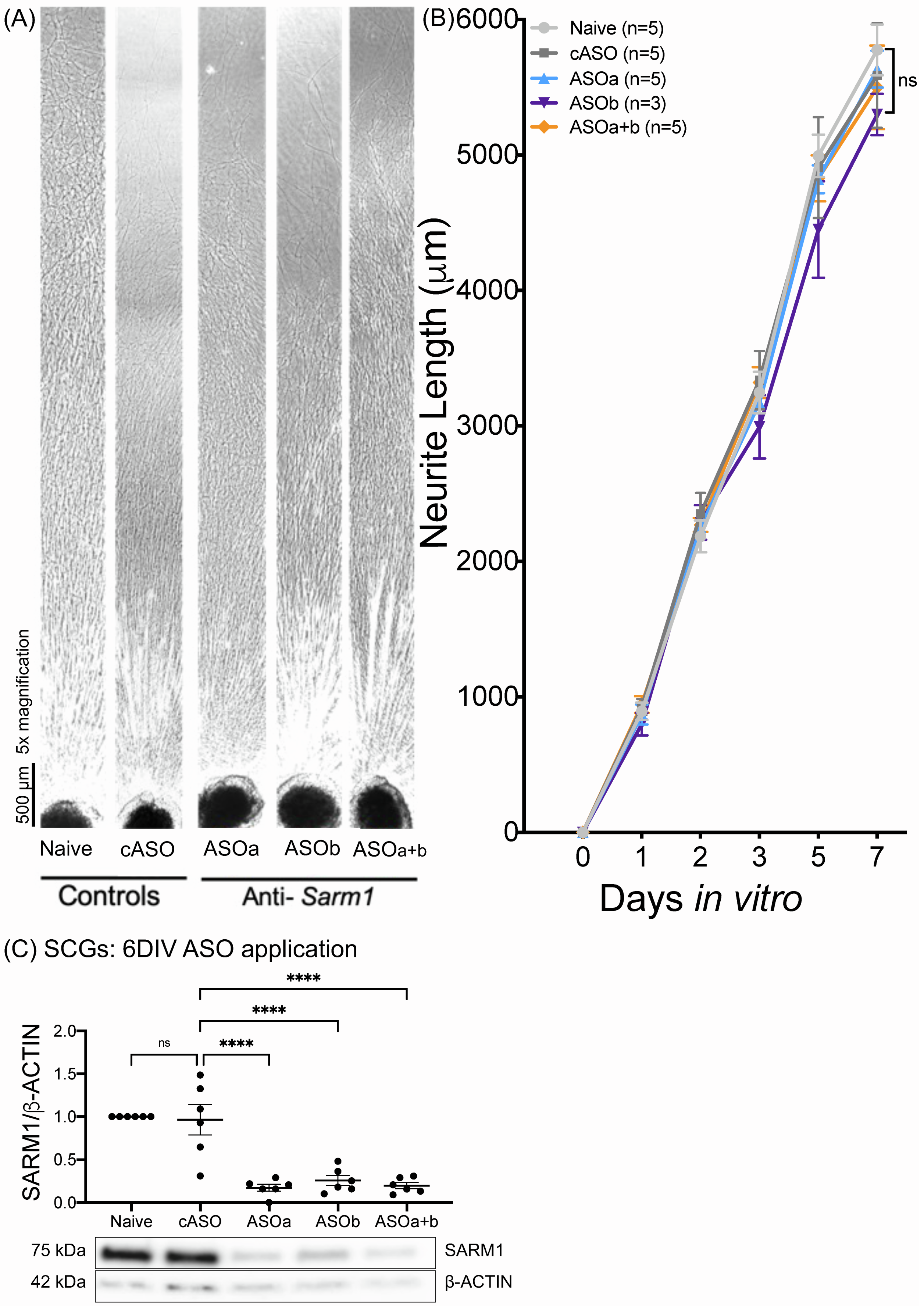

(A)
5x magnification
500 µm
Naive
cASO
ASOa
ASOb
ASOa+b
Controls
Anti- *Sarm1*
(B)
Naive (n=5)
cASO (n=5)
ASOa (n=5)
ASOb (n=3)
ASOa+b (n=5)
ns
Neurite Length (µm)
6000
5000
4000
3000
2000
1000
0
0
1
2
3
5
7
Days *in vitro*
(C) SCGs: 6DIV ASO application
ns
****
****
****
SARM1/β-ACTIN
2.0
1.5
1.0
0.5
0.0
Naive
cASO
ASOa
ASOb
ASOa+b
75 kDa
42 kDa
SARM1
β-ACTIN

Supplemental Figure 1: *Sarm1* antisense oligonucleotides do not affect SCG neurite outgrowth and significantly decrease SARM1 protein levels. Extra controls related to Figures 4 and 5. Representative phase contrast images of full length (5x magnification) SCG explants from P0 mice (a). Quantification of wild-type neurite outgrowth in the presence of plain media (naïve), cASO, ASOa, ASOb, or combined ASOa+b across seven days *in vitro* (b). ASOs were applied the day after plating (day1). Western blot shows that SARM1 band intensity is lower in *Sarm1* ASO-treated SCG cultured for 7DIV than cASO-treated SCGs when normalised to β-ACTIN; representative bands are shown below the quantification (c). A two-way ANOVA was performed followed by Dunnett's post-hoc analysis to determine if there was a significant difference in neurite outgrowth between cASO- and *Sarm1* ASO-treated SCGs. A one-way ANOVA was performed followed by Dunnett's post-hoc analysis to determine whether there was a significant difference in SARM1 band intensity between cASO-treated SCGs and *Sarm1* ASO-treated SCGs. ****$p<0.0001$; ns=not significant. Sample sizes for outgrowth and Western blot were from 3-5 mice per condition. Each data point represents an individual mouse where both SCGs were cultured in the presence of ASOs, or the average neurite length from three fields of view from two SCGs cultured together from one mouse. Data are presented as mean $\pm$ SEM. Scale bar represents 500 µm.

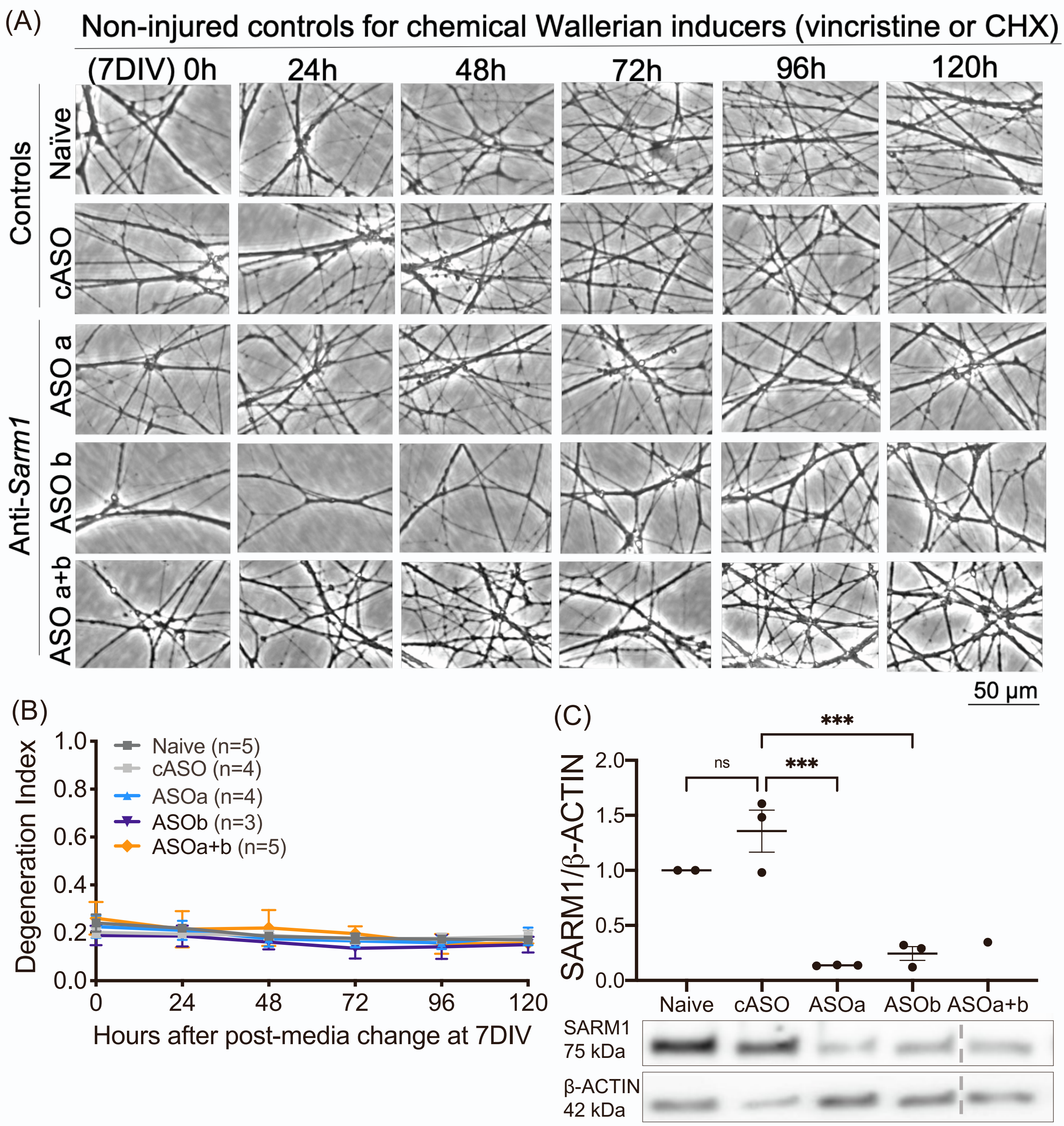
(A)
Non-injured controls for chemical Wallerian inducers (vincristine or CHX)
(7DIV) 0h
24h
48h
72h
96h
120h
Controls
Naïve
cASO
Anti-Sarm1
ASO a
ASO b
ASO a+b
50 μm
(B)
Degeneration Index
Naive (n=5)
cASO (n=4)
ASOa (n=4)
ASOb (n=3)
ASOa+b (n=5)
1.0
0.8
0.6
0.4
0.2
0.0
0
24
48
72
96
120
Hours after post-media change at 7DIV
(C)
SARM1/β-ACTIN
2.0
1.5
1.0
0.5
0.0
ns
***
***
Naive
cASO
ASOa
ASOb
ASOa+b
SARM1
75 kDa
β-ACTIN
42 kDa

Supplemental Figure 2: Prolonged exposure to *Sarm1* antisense oligonucleotides does not impair axon morphology or further lower SARM1 levels. Extra controls related to Figure 5. Representative phase contrast images of distal SCG neurites (20x magnification) from P0 mice (a) and quantification of degeneration index (b). SCGs were grown for 12DIV with ASOs applied for 11DIV (starting the day after plating). At 7DIV (0h), chemical inducers of programmed axon degeneration were applied to experimental groups, but plain media containing ASOs were applied to this cohort to confirm that prolonged exposure did not cause spontaneous degeneration or further lower SARM1 levels. Western blot analysis shows that SARM1 levels are lower in *Sarm1* ASO-treated SCGs than cASO-treated SCGs when normalised to β-ACTIN, but no further decreased than after 6DIV application (compare with S2); representative bands are shown below the quantification; grey line indicates sample was in a non-adjacent well on the same blot (c). A one-way ANOVA was performed followed by Dunnett's post-hoc analysis to determine whether there was a significant difference in SARM1 band intensity between cASO-treated SCGs and *Sarm1* ASO-treated SCGs. ***$p<0.001$. Sample sizes for degeneration index and Western blot were from 1-3 mice per condition, as indicated. Each data point represents an average value from two fields of view from two individual SCGs cultured in the same dish (a-b) or an individual mouse where both SCGs were cultured for 12 days in the presence of ASOs for 11DIV (c). Data are presented as mean ± SEM. Scale bar represents 50 μm.

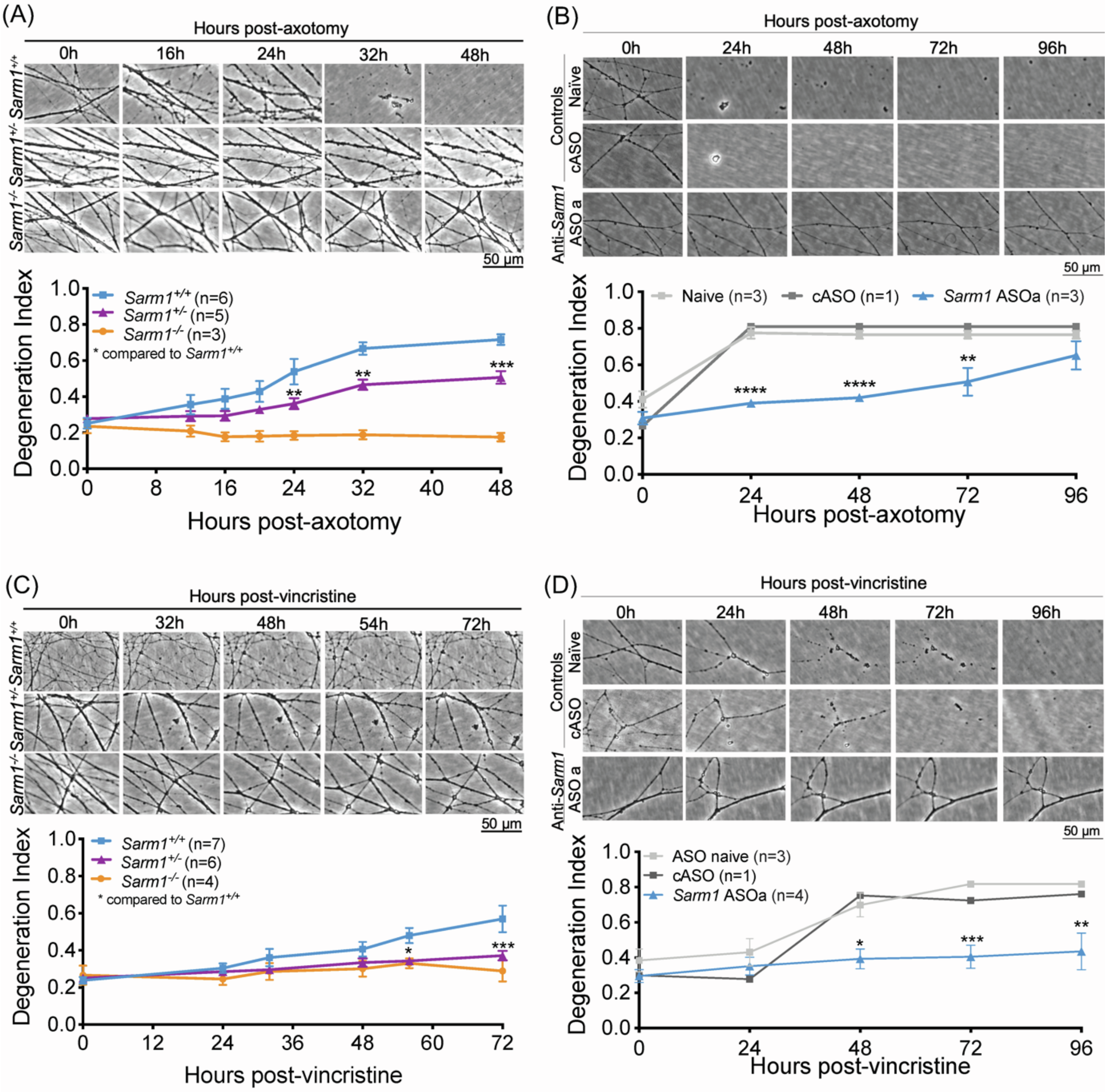
(A)
Hours post-axotomy
0h
16h
24h
32h
48h
Sarm1+/+
Sarm1+/-
Sarm1-/-
50 µm
Degeneration Index
Sarm1+/+ (n=6)
Sarm1+/- (n=5)
Sarm1-/- (n=3)
* compared to Sarm1+/+
Hours post-axotomy
(B)
Hours post-axotomy
0h
24h
48h
72h
96h
Controls
Naïve
cASO
Anti-Sarm1 ASO a
50 µm
Naive (n=3)
cASO (n=1)
Sarm1 ASOa (n=3)
Degeneration Index
Hours post-axotomy
(C)
Hours post-vincristine
0h
32h
48h
54h
72h
Sarm1+/+
Sarm1+/-
Sarm1-/-
50 µm
Degeneration Index
Sarm1+/+ (n=7)
Sarm1+/- (n=6)
Sarm1-/- (n=4)
* compared to Sarm1+/+
Hours post-vincristine
(D)
Hours post-vincristine
0h
24h
48h
72h
96h
Controls
Naïve
cASO
Anti-Sarm1 ASO a
50 µm
Degeneration Index
ASO naive (n=3)
cASO (n=1)
Sarm1 ASOa (n=4)
Hours post-vincristine

Supplemental Figure 3: *Sarm1* haploinsufficiency or antisense oligonucleotides also delay programmed axon degeneration in DRGs. Confirmation of SCG phenotypes seen in Figures 3 and 5 using DRGs. *Sarm1* haploinsufficient, wild-type, or homozygous null DRGs were cultured for 7DIV and then received either axotomy (a) or 20 nM vincristine (c) and the same fields of view were repeatedly imaged at 20x magnification in phase contrast across the indicated time-course. DRGs receiving plain media or ASOa were cultured for 7DIV with ASOs. At 7DIV, cultures received either axotomy (b) or 20 nM vincristine (d) and the same fields of view were repeatedly imaged at 20x magnification in phase contrast across the indicated time-course. Representative micrographs for each genotype or ASO-treatment group at each timepoint are shown above quantification of axon degeneration. A two-way repeated measures ANOVA was performed followed by Bonferroni post-hoc analysis to determine whether there was a difference between *Sarm1* haploinsufficient and wild-type or homozygous null DRGs (a and c) or Dunnett's post-hoc analysis to determine whether there was a difference or Naive and *Sarm1* ASOa-treated DRGs (b and d). *$p<0.05$; **$p<0.01$; ***$p<0.001$; ****$p<0.0001$. Sample sizes are indicated on graphs for each condition. Each data point represents an average value from three fields of view from three individual DRGs cultured in the same dish. Data are presented as mean $\pm$ SEM. Scale bars represent 50 µm.
